# Gut Microbial Genes and Metabolism for Methionine and Branched-Chain Amino Acids in Diabetic Nephropathy

**DOI:** 10.1128/spectrum.02344-22

**Published:** 2023-03-06

**Authors:** Ji Eun Kim, Hoonsik Nam, Ji In Park, Hyunjeong Cho, Jangwook Lee, Hyo-Eun Kim, Dong Ki Kim, Kwon Wook Joo, Yon Su Kim, Bong-Soo Kim, Sunghyouk Park, Hajeong Lee

**Affiliations:** a Department of Internal Medicine, Korea University Guro Hospital, Seoul, South Korea; b College of Pharmacy, Natural Products Research Institute, Seoul National University, Seoul, South Korea; c Department of Internal Medicine, Kangwon National University Hospital, Chuncheon, South Korea; d Department of Internal Medicine, Chungbuk National University Hospital, Cheongju, South Korea; e Department of Internal Medicine, Dongguk University Ilsan Hospital, Ilsan, South Korea; f Seoul National University Hospital Biomedical Research Institute, Seoul, South Korea; g Kidney Research Institute, Seoul National University, Seoul, South Korea; h Department of Internal Medicine, Seoul National University Hospital, Seoul, South Korea; i Department of Internal Medicine, Seoul National University College of Medicine, Seoul, South Korea; j Department of Life Science, Multidisciplinary Genome Institute, Hallym University, Chuncheon, South Korea; Lerner Research Institute

**Keywords:** branched-chain amino acid, diabetic nephropathy, metabolite, metagenome, methionine, microbiota, gut microbiota

## Abstract

Diabetic mellitus nephropathy (DMN) is a serious complication of diabetes and a major health concern. Although the pathophysiology of diabetes mellitus (DM) leading to DMN is uncertain, recent evidence suggests the involvement of the gut microbiome. This study aimed to determine the relationships among gut microbial species, genes, and metabolites in DMN through an integrated clinical, taxonomic, genomic, and metabolomic analysis. Whole-metagenome shotgun sequencing and nuclear magnetic resonance metabolomic analyses were performed on stool samples from 15 patients with DMN and 22 healthy controls. Six bacterial species were identified to be significantly elevated in the DMN patients after adjusting for age, sex, body mass index, and estimated glomerular filtration rate (eGFR). Multivariate analysis found 216 microbial genes and 6 metabolites (higher valine, isoleucine, methionine, valerate, and phenylacetate levels in the DMN group and higher acetate levels in the control group) that were differentially present between the DMN and control groups. Integrated analysis of all of these parameters and clinical data using the random-forest model showed that methionine and branched-chain amino acids (BCAAs) were among the most significant features, next to the eGFR and proteinuria, in differentiating the DMN group from the control group. Metabolic pathway gene analysis of BCAAs and methionine also revealed that many genes involved in the biosynthesis of these metabolites were elevated in the six species that were more abundant in the DMN group. The suggested correlation among taxonomic, genetic, and metabolic features of the gut microbiome would expand our understanding of gut microbial involvement in the pathogenesis of DMN and may provide potential therapeutic targets for DMN.

**IMPORTANCE** Whole metagenomic sequencing uncovered specific members of the gut microbiota associated with DMN. The gene families derived from the discovered species are involved in the metabolic pathways of methionine and branched-chain amino acids. Metabolomic analysis using stool samples showed increased methionine and branched-chain amino acids in DMN. These integrative omics results provide evidence of the gut microbiota-associated pathophysiology of DMN, which can be further studied for disease-modulating effects via prebiotics or probiotics.

## INTRODUCTION

Diabetes mellitus (DM) is a common metabolic disorder that causes a significant medical burden worldwide. The global prevalence of DM is substantially increasing and is expected to increase by over 10% in 2045 (1). In South Korea, the prevalence of DM among adults aged ≥30 years has increased to 13.8% (2). About 25 to 40% of patients with DM develop diabetic mellitus nephropathy (DMN), a major microvascular complication of DM. DMN is the leading cause of chronic kidney disease (CKD) and end-stage kidney disease (ESKD) (3–5). It is characterized by chronic glomerular filtration barrier dysfunction (represented by albumin leakage to urine), subsequent structural or functional deterioration of the kidney, and, ultimately, progression to ESKD. Patients with DMN show a higher risk of cardiovascular diseases and all-cause mortality than those with either diabetes or CKD alone (6–8). Owing to the complex and uncertain pathogenesis of DMN, the prevention of DMN occurrence or progression remains unresolved.

The gut microbiota coexists with humans, and its metabolites play pivotal roles in host homeostasis and disease development (9). For example, an imbalance of the gut microbial composition, known as dysbiosis, is associated with many chronic diseases (10–14). DMN is also characterized by an altered intestinal microbiota (15–17). Gut metagenome profiles associated with tyrosine and butyrate production are altered in type 2 DM compared with those in controls (18). Microbiome-associated markers can distinguish between DMN and membranous nephropathy (19). In addition, functional changes in the gut metagenome that produce specific metabolites of lipid, amino acid, tricarboxylic acid cycle and urea cycle in DMN have been reported (20–23). Nevertheless, there is a lack of integrated research with metabolites actually measured in feces combined with changes in the functional pathways and gene families associated with the microbiota rather than simple differences in microbiota abundance.

This study aimed to identify coherent microbial factors that could differentiate healthy individuals from DMN patients using an integrated analysis of clinical information and stool metagenomic and metabolomic data. The present study provides a fundamental basis for understanding pathological and biochemical changes in DMN.

## RESULTS

### Baseline characteristics.

Fifteen patients with clinicopathologically diagnosed DMN and 22 healthy controls were studied. Comparing the baseline characteristics of the two groups, we found that the DMN group had an older age (*P* = 0.023), a higher proportion of male patients (*P* = 0.001), and a higher body mass index (BMI) (*P* = 0.183) than the control group. The mean HbA1c levels were 7.1% ± 1.3% and 5.4% ± 0.3% in the DMN and the control groups, respectively. The DMN group showed a higher level of proteinuria and a lower estimated glomerular filtration rate (eGFR) than the controls. In the DMN group, 41.7% of the patients were administered insulin as a treatment agent, and 73.3% of the patients were prescribed one or more types of oral hypoglycemic agents. The mean duration of DM was 13.7 ± 6.7 years. The detailed clinical characteristics of the groups are shown in Table 1.

**TABLE 1:** Baseline characteristics of the participants^*a*^

Variable	Value for group		*P* value
	DMN (*n* = 15)	Healthy controls (*n* = 22)	
Mean age (yrs) ± SD	56.8 ± 11.1	51.8 ± 9.6	0.023
% male patients	86.7	31.8	0.001
Mean BMI (kg/m^2^) ± SD	25.7 ± 4.0	23.7 ± 2.5	0.183
Mean eGFR (mL/min/1.73 m^2^) ± SD	48.4 ± 22.7	103.1 ± 9.7	<0.001
Mean urine protein/creatinine ratio (g/g) ± SD	6.3 ± 4.7	0.05 ± 0.04	<0.001
Mean % HbA1c ± SD	7.1 ± 1.3	5.4 ± 0.3	<0.001
Mean glucose concn (mg/dL) ± SD	143.3 ± 109.7	107.4 ± 16.8	0.899
Mean duration of diabetes mellitus (yrs) ± SD	13.7 ± 6.7	NA	NA
% of patients taking glucose-lowering agent			
Insulin	46.7	NA	NA
Metformin	46.7	NA	NA
Sulfonylurea	46.7	NA	NA
DPP4 inhibitor	60	NA	NA

a DMN, diabetic nephropathy; BMI, body mass index; eGFR, estimated glomerular filtration rate; DPP4, dipeptidyl peptidase 4; NA, not available.

### Differences in taxonomic and functional gene features in the gut microbiome between the DMN and control groups.

The total number of reads in the shotgun analysis was 6,099,635 (range, 5,178,717 to 6,959,188). The DMN and control groups showed 6,085,912 (range, 5,339,071 to 7,509,547) (78.5% [range, 76.5 to 81.4%] of the total reads after removing human genes) and 6,102,815 (range, 5,175,165 to 6,959,188) (82.1% [range, 81.1 to 83.8%] of the total reads after removing human genes) microbiota gene reads, respectively. Rarefaction curves for each sample are plotted in Fig. S1 in the supplemental material.

**FIG 1:**
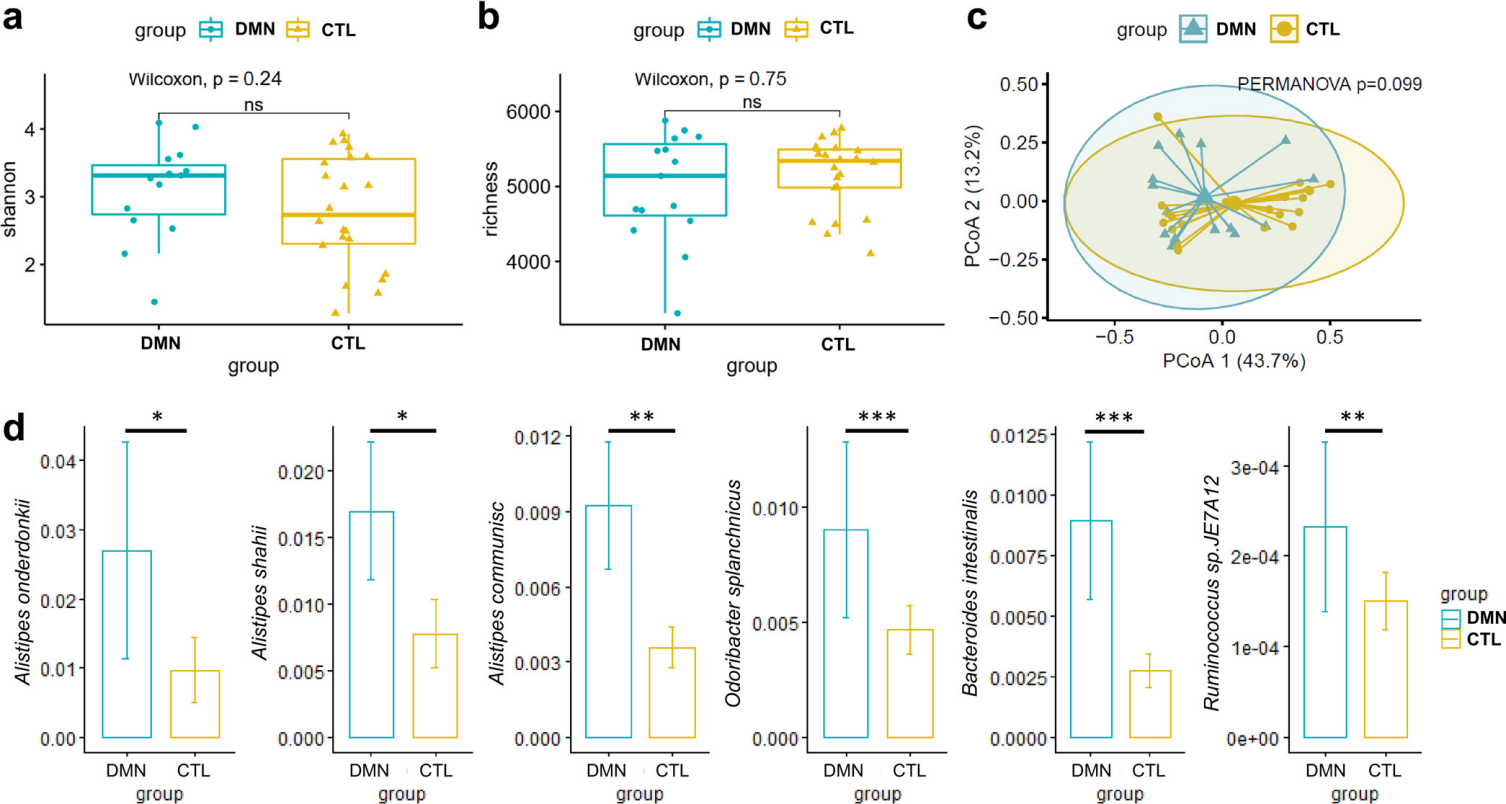
Gut microbial differences between the diabetic mellitus nephropathy (DMN) and healthy control (CTL) groups. (a to c) Shannon diversity (a), richness (b), and principal-coordinate analysis (PCoA) (c) plots calculated with the Bray-Curtis method for the DMN and healthy control groups. *P* values for alpha diversity and beta diversity metrics were calculated by a Wilcoxon rank sum test and PERMANOMA, respectively. ns, not significant. (d) Six species showed significant differences between the DMN and healthy control groups by multivariable-adjusted MaAsLin2 analysis (*, *P* < 0.05; **, *P* < 0.01; ***, *P* < 0.001).

The Shannon diversity index and richness of the gut microbiota were similar in the DMN and control groups (Fig. 1a and b). However, in the principal-coordinate analysis without adjustment for clinical characteristics, the microbiota compositions were not statistically different between the two groups (Fig. 1c). Focusing on the 79 species with an average relative abundance of >0.1% and the variable control for age, sex, BMI, and eGFR, Multivariate Association with Linear Models (MaAsLin2) analysis identified six species that were significantly different between DMN patients and controls (Fig. 1d). These 6 species exhibited elevated relative abundances in DMN patients compared to controls. These species were Alistipes onderdonkii (*P *= 0.004; *q *= 0.071), Alistipes shahii (*P *= 0.004; *q *= 0.071), Alistipes communis (*P *= 0.002; *q *= 0.044), *Ruminococcus* sp. strain JE7A12 (*P *= 0.002; *q *= 0.044), Bacteroides intestinalis (*P < *0.001; *q *= 0.006), and Odoribacter splanchnicus (*P < *0.001; *q *= 0.003).

**FIG 2:**
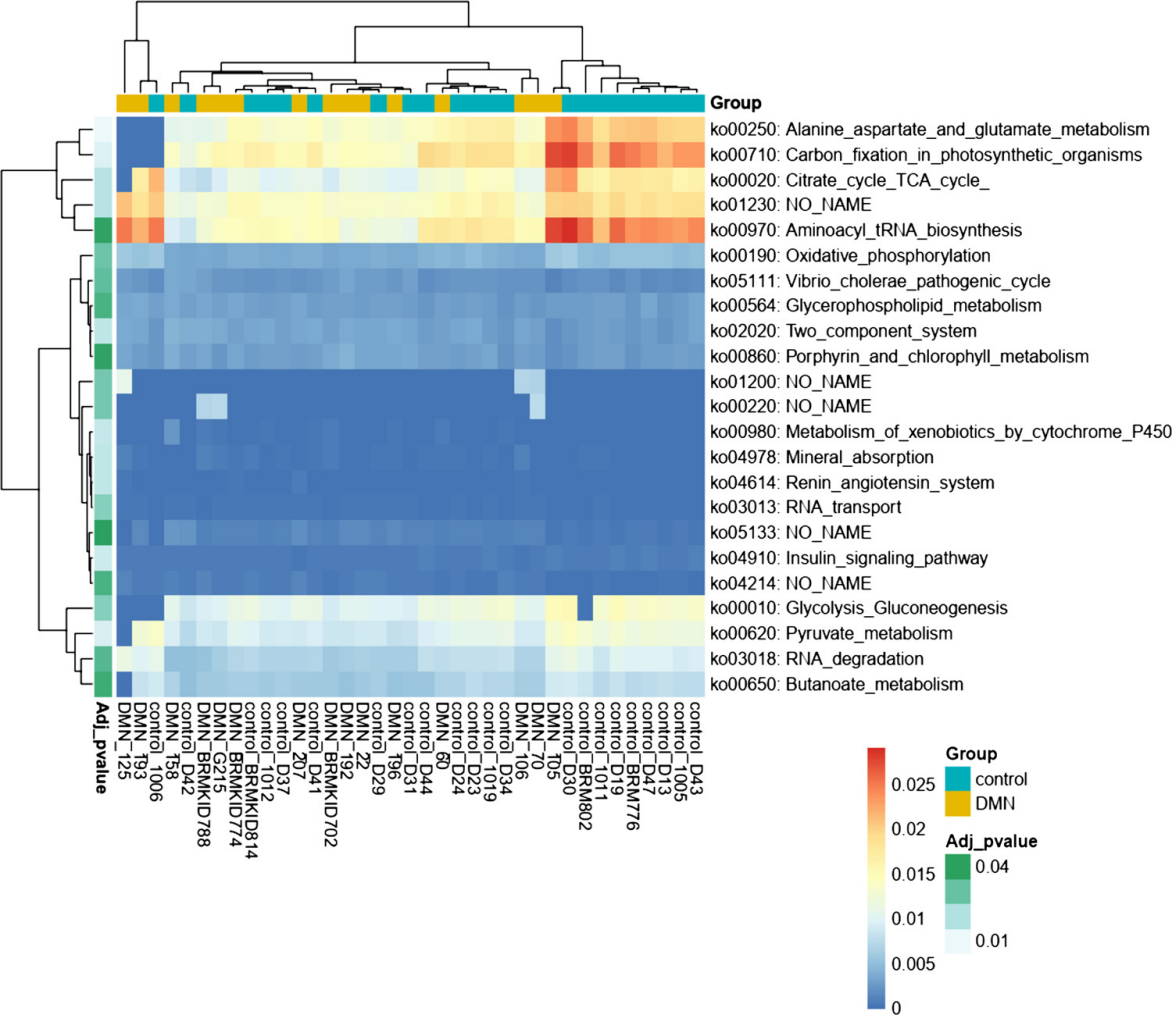
Heat map plot of functional pathways. Significantly different functional KEGG pathways between the diabetic mellitus nephropathy (DMN) and healthy control (CTL) groups are listed in the rows. Sample names are listed in the columns. Colors in the heat map represent the abundance of each pathway in samples. Adjusted *P* values and group classifications are shown at the left and top margins of the heat map, respectively. TCA, tricarboxylic acid.

In the functional pathway analysis using the Kyoto Encyclopedia of Genes and Genomes (KEGG) database, 24 functional pathways were differentially expressed in DMN patients compared with the controls. Among them, 11 pathways, such as alanine, aspartate, and glutamate metabolism (ko00250); pyruvate metabolism (ko00620); the citrate cycle (ko00020); and glycolysis/gluconeogenesis (ko00010), were included in the metabolism pathway (ko09100). In DMN patients, decreased functional pathways for glycolysis/gluconeogenesis, pyruvate metabolism, and the citrate cycle represent altered glucose metabolism. Furthermore, decreased alanine, aspartate, and glutamate metabolism may reflect an alteration of branched-chain amino acid (BCAA) synthesis by changing α-ketoglutarate, the main accepter of the amino nitrogen of BCAAs, in diabetes. Significantly different pathways between groups are listed in a heat map plot (Fig. 2).

Of the 6,179 KEGG-assigned gene families, 216 genes involved in metabolism (ko09100) were significantly different between DMN patients and controls by multivariable analysis using MaAsLin2 (*q *< 0.25) (Table S1). They included 54, 43, and 13 genes for carbohydrate, protein, and lipid metabolism (ko09101, ko09105, and ko09103), respectively. Among the 54 carbohydrate metabolism genes, 11 were related to either glycolysis or gluconeogenesis. All significantly different gene families, including carbohydrate, protein, and lipid metabolisms, were elevated in DMN patients compared to controls (Table S2).

### Stool metabolite differences between the DMN and control groups.

**FIG 3:**
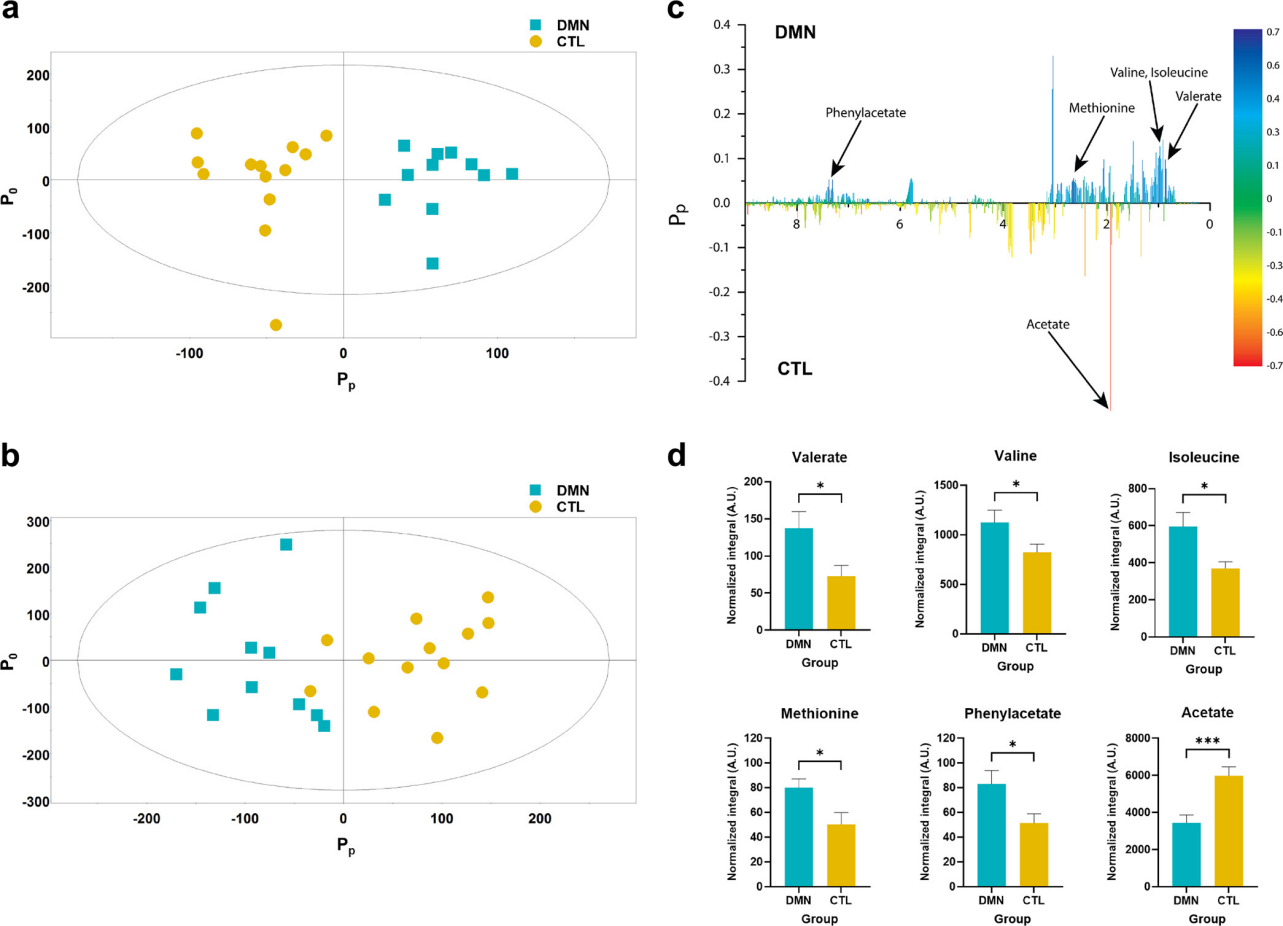
Stool metabolite profiling in the diabetic mellitus nephropathy (DMN) and healthy control (CTL) groups. (a and b) OPLS-DA score plots for water-soluble (a) and lipid-soluble (b) metabolites of the DMN and healthy control groups. Models were obtained using one predictive (*P_p_*) and two orthogonal (*P_o_*) components (*n* = 11 for the DMN group; *n* = 13 for the healthy control group). (c) Metabolites contributing to DMN from statistical total correlation spectroscopy (STOCSY). The model coefficients for each NMR variable from water-soluble metabolites are shown. As a discriminator between the two groups, a color scale based on the value of *P*_(_*_corr_*_)_*_p_* according to weight is used. *P_p_* represents the modeled covariant. Metabolite signals that differed significantly between the DMN and healthy control groups are depicted on the coefficient plot. (d) Levels of the six metabolites are significantly different between the DMN and healthy control groups. The levels represent binned peak integrals from the NMR spectrum normalized against the sum of the integrals of the entire spectrum. All bar plots and error bars represent means and standard errors, respectively (*, 0.01 < *P* < 0.05; ***, *P* < 0.001). A.U., arbitrary units.

Of the 37 participants, stool metabolites from 24 participants (11 DMN patients and 13 controls) in both the water-soluble and lipid-soluble fractions were assessed by nuclear magnetic resonance (NMR) spectroscopy. Raw NMR data were analyzed by multivariate orthogonal projections to latent structures discriminant analysis (OPLS-DA). The resulting score plots discriminating the DMN and control groups were obtained with one predictive component (*P_p_*) and two orthogonal components (*P_o_*) (Fig. 3a and b). For water-soluble metabolites, the OPLS-DA model showed good separation between the DMN and control groups (*R*^2^ = 0.852; *Q*^2^ = 0.566), while the goodness of fit and predictive values were lower for the lipid-soluble metabolites (*R*^2^ = 0.706; *Q*^2^ = 0.296). Therefore, the major metabolites that differentiate the two groups were identified from the water-soluble metabolome using statistical total correlation spectroscopy (STOCSY) (Fig. 3c). This analysis revealed that the levels of valine, isoleucine, methionine, valerate, and phenylacetate were significantly higher and that the level of acetate was lower in the DMN group (Fig. 3d).

### Integrated feature selection related to DMN using clinical information, bacterial species, genes, and stool metabolites.

**FIG 4:**
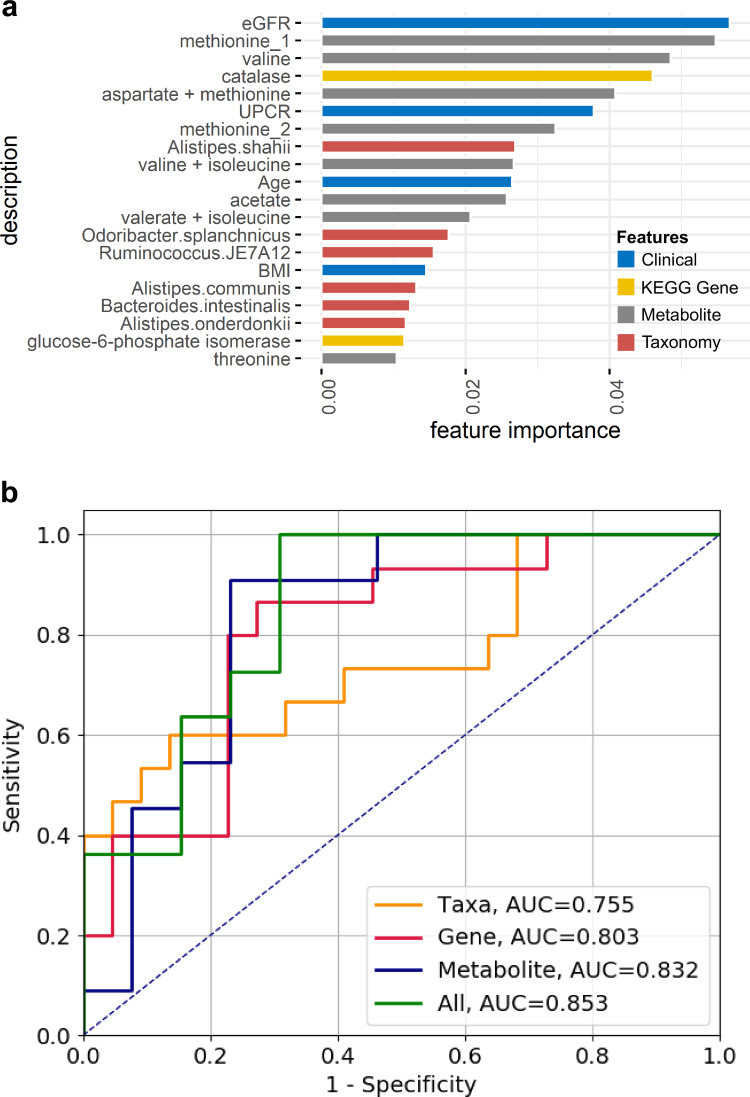
Integrated analysis of the predictive potential of variables for DMN. (a) Feature importance values of the top 20 variables obtained from random-forest analysis on clinical factors (blue), KEGG gene families (yellow), stool metabolites (gray), and gut microbiota species (red). Clinical factors included in this analysis were age, sex, BMI, eGFR, and UPCR. KEGG gene families and microbiota species that showed significant results in the MaAsLin2 analysis and stool metabolites found using peak-level differences in the NMR study are included. (b) Receiver operating characteristic curves for the prediction of DMN. The *C* statistics were calculated from all conclusive taxonomy, genes, and metabolites (green); taxonomy alone (yellow); KEGG gene family alone (red); and stool metabolites alone (blue). AUC, area under the curve.

Among the differentially present clinical, metagenomic, and metabolomic features between the DMN and control groups, random-forest analysis identified the top 20 most important features. As expected, the traditional clinical characteristics of DMN, a low eGFR and a high level of proteinuria, ranked as the top features. The six significant stool species and gene families for catalase and glucose-6-phosphate isomerase were also listed among the top features. Eight of the top 20 features were the peak assignment values for specific metabolites. Moreover, the feature importance scores for fecal methionine, valine, and aspartate were close to those for the clinical eGFR and urine protein-to-creatinine ratio (UPCR) parameters (Fig. 4a). The discriminating power of each category was assessed using *C* statistics. The area under the curve values obtained for all three categories (species, gene pathways, and stool metabolites [0.853]) were higher than those obtained for individual categories (Fig. 4b).

### Microbial genes for methionine and branched-chain amino acid synthesis.

**FIG 5:**
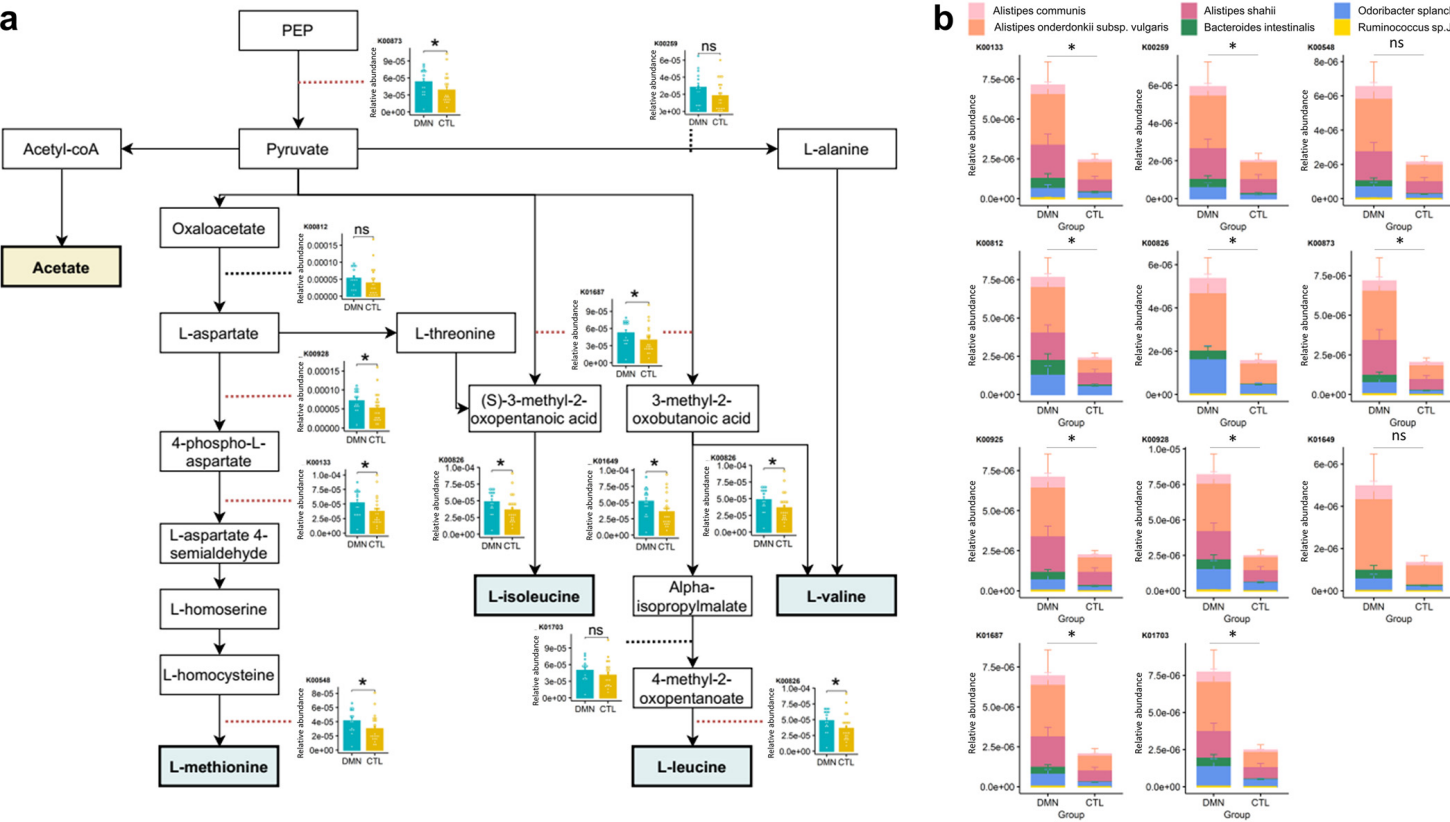
(a) Pathways and gene families related to the biosynthesis of methionine and BCAAs. Blue and yellow squares represent dominant metabolites in the diabetic mellitus nephropathy (DMN) and healthy control (CTL) groups, respectively. Bar plots represent differences in each gene between the DMN and healthy control groups. All genes with bar plots were significantly different in the multivariable-adjusted MaAsLin2 analysis, and the asterisks in the bar plots represent *P* values of <0.05 by Kruskal-Wallis tests. PEP, phosphoenolpyruvate. (b) Contribution of specific members of the gut microbiota to the significant genes associated with methionine and BCAA pathways. All bar plots and error bars represent means and standard errors, respectively.

As we found that methionine as well as BCAAs, including valine and isoleucine, were important features of DMN in the NMR analysis, the relationships among the members of the stool microbiome, their functional genes, and the metabolites were further assessed in the context of methionine and BCAA metabolism. Of the significant metabolism-related genes found in the multivariate analysis (Table S2), the levels of genes related to the biosynthesis of aspartate, methionine, and BCAAs were elevated in the DMN patients compared to the controls. For example, the levels of genes of the pyruvate-to-methionine synthesis pathway, including *aspB*, *lysC*, *asd*, and *metH*, were significantly elevated in DMN patients (*P* values of 0.011, 0.006, 0.002, and <0.001, respectively, by MaAsLin2 analysis; *P* values of 0.101, 0.038, 0.038, and 0.041, respectively, by Kruskal-Wallis analysis). Similarly, the levels of genes of the BCAA synthesis pathway, such as *ilvD*, *ilvE*, *leuA*, *leuC*, and *ald*, were also elevated in the DMN patients (*P* values of <0.001, 0.003, <0.001, 0.006, and <0.001, respectively, by MaAsLin2 analysis; *P* values of 0.044, 0.024, 0.028, 0.101, and 0.078, respectively, by Kruskal-Wallis analysis) (Fig. 5a). The relative contributions of the six bacterial species to these metabolic genes were significantly different between the DMN and control groups, suggesting that the changes in the gut microbiome may have elevated the stool metabolites in DMN (Fig. 5b).

### Correlation between stool metabolites and clinical severity of diabetes.

**FIG 6:**
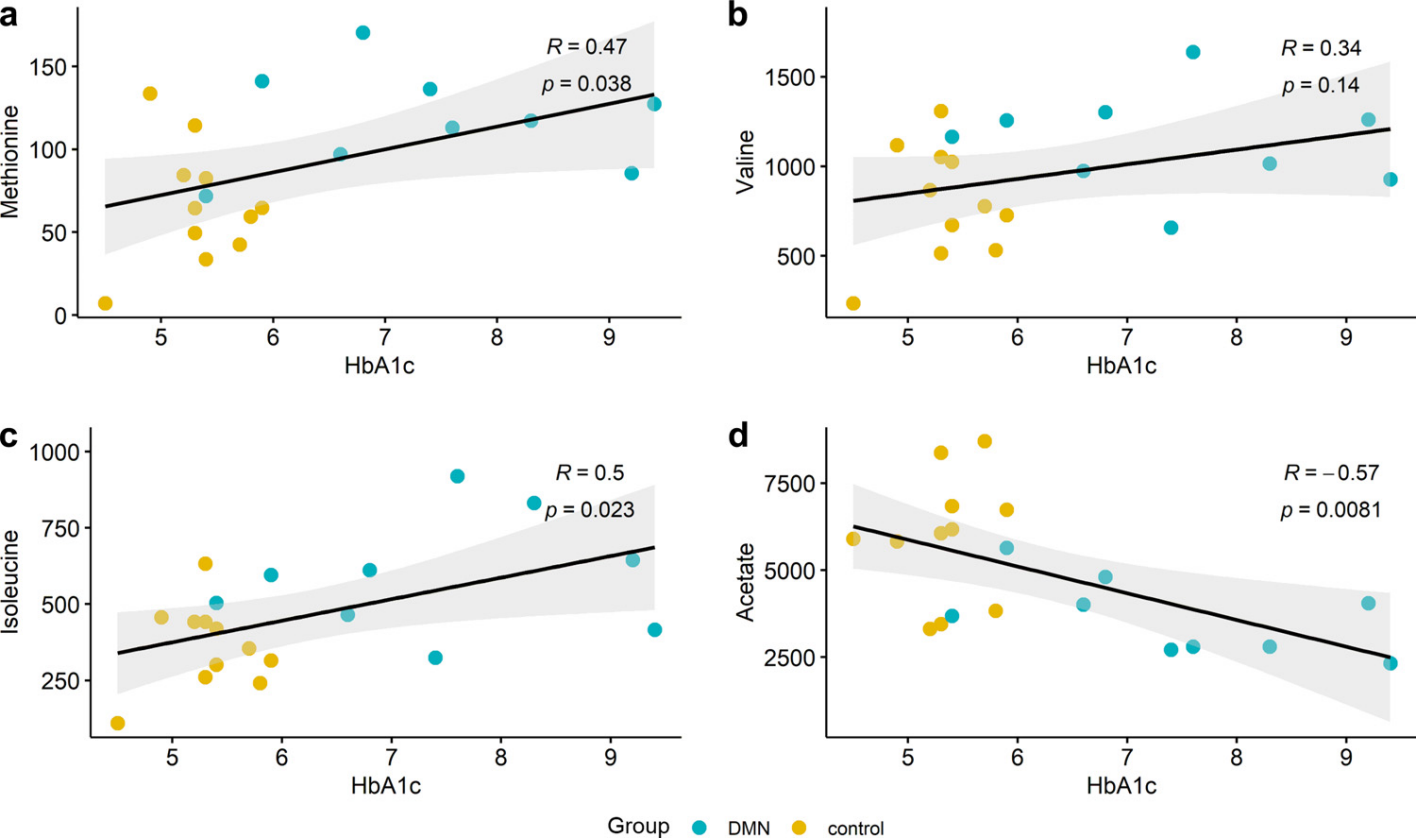
Association between HbA1c and stool metabolites. (a to c) Among methionine, valine, and isoleucine, which are significantly elevated in DMN, methionine and isoleucine showed a significant linear association with HbA1c levels. (d) Acetate, which was decreased in DMN, showed a reverse association with HbA1c levels. Black solid lines and gray zones in each graph represent fitted regression lines and 95% confidence intervals, respectively.

HbA1c levels are usually monitored to track and determine the severity of glucose dysregulation in diabetes. Thus, we analyzed the correlation between HbA1c and significant stool metabolites to determine whether these metabolites are associated with disease severity (Fig. 6). Linear regression analyses showed that the stool methionine and isoleucine levels were positively correlated, the stool valine levels were not significantly correlated, and the stool acetate levels were negatively correlated with HbA1c levels.

## DISCUSSION

We found that several fecal metabolites, especially methionine and BCAAs, are present differently between the DMN and control groups and are associated with gut microbes, including *Alistipes* spp. Integrated taxonomic, genetic, and metabolic analyses suggested the possibility that dysbiosis could regulate the synthesis and degradation of metabolites and cause DM-associated complications. The metabolic roles of the gut microbiome have recently been recognized (24, 25), which may have effects on various disease conditions and immunoregulation (26–28).

Recent studies have reported gut microbiome-associated advances in the diagnosis and pathogenesis of DM and DMN (11, 29–32). In a 16S rRNA sequencing-based study comparing membranous nephropathy and DMN, the *Blautia* and *Akkermansia* genera were significantly abundant in DMN patients (19). In a whole-metagenome sequencing analysis, a mathematical model based on metagenomic profiles was developed to diagnose type 2 DM in European women (16). Another whole-metagenome study showed that the levels of 51 species of potential butyrate producers were significantly lower in DM patients than in healthy controls (33). In the present study, whole-metagenome analysis revealed that six bacterial species, A. onderdonkii, A. shahii, A. communis, B. intestinalis, O. splanchnicus, and *Ruminococcus* sp. JE7A12, were more abundant in fecal samples of DMN patients. However, previously reported findings on these species in terms of their association with DM or DMN are inconsistent. The *Alistipes* genus was reported to be a detrimental factor in DMN and positively associated with proteinuria (34). In contrast, a whole-metagenome analysis of European women revealed that *A. shahii* was elevated in the healthy control group compared to the diabetes group (16). There are several reasons for the differences observed between our study and previous studies. We used a multivariate-adjusted analysis to compensate for the effects of age, sex, and BMI on the gut microbiome. In addition, ethnic, dietary, and geographical differences may have contributed to the differences observed in DMN-associated gut microbial species (35, 36).

Regarding the gene-metabolite association in DMN, our study revealed that stool methionine and BCAA levels were higher in the DMN group. Differences in the microbial genes involved in the biosynthetic pathways of these metabolites were also observed between the DMN and control groups. By functional pathway analysis of the metagenome, we found that multiple pathways associated with glycolysis were significantly decreased in diabetic nephropathy. Decreased glycolysis and preferential use of fatty acids may result in decreased flux through the citric acid cycle and a decreased supply of amino group acceptors (α-ketoglutarate, pyruvate, and oxaloacetate) for BCAA aminotransferase and alanine and aspartate transaminase reactions, subsequently leading to excessive BCAA production (37, 38). Methionine, a sulfur-containing amino acid, is converted intracellularly to homocysteine through transmethylation with a methyl acceptor (39). Homocysteine can accumulate in the blood as well as intracellular fluid in various disease conditions, including cardiovascular complications, obesity, and type 2 diabetes with kidney insufficiency and/or proteinuria, with suspicion of the association between insulin and methionine/homocysteine metabolism (40, 41). In addition, dietary methionine restriction may prevent incident diabetes mellitus in diabetes-prone mice (42). Higher plasma BCAA levels have been associated with insulin resistance or diabetes (43, 44). Both BCAAs and methionine, which seem to be associated with DM and associated complications, are essential amino acids; they cannot be synthesized *de novo* by human genes, and therefore, they should be supplied and absorbed from food, suggesting a biological link with the gut microbiota (45). However, there have been only a few human studies that have shown an association between fecal methionine or BCAAs and DM or DMN (20, 46, 47). Most studies showed elevated methionine or BCAA levels in diabetic patients without microbiota evaluation. Our results regarding BCAAs and associated genes were not only consistent with those of previous studies, but also propose associated members of the gut microbiota related to these genes. We provide evidence of the presence of genes related to BCAAs from specific members of the microbiota abundant in diabetic nephropathy, but additional metatranscriptomic research is needed for the evaluation of actual gene expression.

This study on integrated taxonomic, functional gene, and metabolite analyses with the adjustment of various clinical factors showed results supporting those of previous reports. However, this study has several limitations. This study may provide correlations among clinical factors, the metagenome, and stool metabolites but cannot suggest a causal relationship among them. We measured only the metabolites in stool; the metabolite levels in circulation or the effects of metabolites on specific organs were not determined in this study. In addition, the numbers of participants in the two groups were small. The different clinical characteristics might also have affected the results. Finally, the present study focused only on the highly predictive features of DMN and represented mainly specific genes in pathways associated with key predictive metabolites. Therefore, the importance of other pathways might have been underestimated. Considering these overall limitations, this study should be regarded as preliminary research for the further integrative study of the metagenome and metabolome.

Here, we highlight the association of microbiota metabolites with DMN development. This suggests the roles of the members of the gut microbiota and their metabolism as a conceptual approach to human DMN pathophysiology. Therefore, further research is warranted to discover effective ways to prevent and treat DM and its associated complications.

## MATERIALS AND METHODS

### Study participants.

This study was approved by the Institutional Review Board (IRB) of the Seoul National University Hospital (IRB approval number 1808-153-967) and complied with the Declaration of Helsinki. Human resources were obtained from the stool repository (IRB approval number 1404-117-575) after the review and approval of the research proposals by the Institutional Review Board of the Seoul National University Hospital. Participants who provided informed consent for genomic analysis and deposition of fecal specimens in the human fecal repository were included.

Patients with biopsy-proven diabetic nephropathy were included in the DMN group. Adults who were identified to be healthy enough to donate their kidneys through a comprehensive medical checkup performed before kidney donation surgery were included in the control group. Data on demographic characteristics, including age, sex, height, and weight, of the DMN and control groups were collected. Laboratory findings relevant to DMN, including serum creatinine, blood HbA1c, serum glucose, random urine protein, and random urine creatinine, were also obtained. The body mass index (BMI) was calculated as body weight (kilograms) divided by height in meters squared. The estimated glomerular filtration rate (eGFR), a marker of kidney function, was calculated using the Chronic Kidney Disease Epidemiology Collaboration calculation formula (48). In the DMN group, the duration of diabetes and the types of glucose-lowering agents used at the time of sample collection were also determined.

### Stool DNA extraction and shotgun sequencing.

The stool samples collected from DMN patients and healthy controls were immediately stored in a deep freezer at −80°C. Stool DNA extraction was performed using a QIAamp Fast DNA stool minikit (Qiagen, Hilden, Germany) according to the manufacturer’s instructions (49, 50). All samples were sequenced on an Illumina NovaSeq 6000 instrument (Illumina, Inc., San Diego, CA, USA). Paired-end reads were generated at 250 bp in the forward and reverse directions.

### Sequence data analysis.

Raw sequence reads were filtered and trimmed with Trimmomatic (version 0.39) using a quality threshold of 20 (51). After merging the paired-end sequences using PEAR (52), contaminating human reads were removed by BBmap 38.87 using the reference human genome (HG19) (53). Taxonomic profiling was performed on filtered reads using Kraken 2 and Bracken (54). Functional profiling was performed using HUMAnN3 (55), applied with a customized Kyoto Encyclopedia of Genes and Genomes (KEGG) database (version 97.0, updated on 1 January 2021) (56, 57). The gene family abundances reported as reads per kilobase were normalized as relative abundances for use in statistical tests.

### Stool metabolite assessment.

Metabolite extraction from stool samples was performed using liquid-liquid extraction. Stool samples stored at −80°C were thawed on ice. Four hundred microliters of methanol and 200 μL of chloroform were added to 200-mg stool samples. The mixture was vortexed for 30 s, frozen in liquid nitrogen for 1 min, and thawed at room temperature. After three freeze-thaw cycles, 200 μL of chloroform and 200 μL of water were added to the samples, and they were again vortexed for 30 s. Finally, the mixtures were centrifuged for 20 min at 18,500 × *g* at 4°C. Water-soluble and lipid-soluble layers of the supernatant were separated and dried for 4 h using a centrifugal vacuum concentrator. Pellets from the water-soluble layer were resuspended in 500 μL nuclear magnetic resonance (NMR) buffer [5 mM NaH_2_PO_4_, 2 mM Na_2_HPO_4_, and 0.025% sodium 3-(trimethylsilyl)propionate-*d*_4_ (TSP) in deuterium oxide (D_2_O)], and pellets from the lipid-soluble layer were resuspended in 500 μL deuterated chloroform (CDCl_3_). Finally, 500 μL of stool extracts was transferred to 5-mm NMR tubes. Proton NMR spectra were acquired with an 800-MHz NMR spectrometer (Avance III; Bruker BioSpin, Germany) operating at an 800.19-MHz proton at 298 K using a one-dimensional (1D) nuclear Overhauser effect spectroscopy (NOESY) pulse sequence (noesypr1d). The proton spectral flame ionization detection free induction decay (FID) data were collected with 32,000 complex data points, a 12-ppm spectral width, and 128 scans.

### NMR data processing and metabolite identification.

The proton NMR spectral data were processed by Fourier transformation, phase correction, baseline correction, and referencing. Spectral data from the water-soluble metabolites were normalized using the internal-standard TSP signal, binned to a width of 0.0147 ppm using an in-house Perl script, and numerically transformed. Spectral data from the lipid-soluble metabolites were normalized against deuterated chloroform and binned to a width of 0.0055 ppm. Spectral regions of TSP (0.0 to 0.2 ppm), glycerol (3.34 to 3.38 and 3.52 to 3.82 ppm), and water (4.7 to 4.9 ppm) were excluded from the analysis of water-soluble metabolites, and the spectral region of chloroform (7.2 to 7.3 ppm) was excluded from the analysis of lipid-soluble metabolites. For metabolite identification, all proton NMR signals were referenced using TSP (for water-soluble metabolites) or chloroform (for lipid-soluble metabolites) signals and identified using the Chenomx NMR suite (Chenomx, Inc., Edmonton, AB, Canada).

### Statistical analysis.

The Shannon index and species richness were calculated using the vegan package in R software version 4.0.3. Principal-coordinate analysis was performed to analyze differences in the microbiota between groups based on the Bray-Curtis distance. The *P* values were calculated by permutational multivariate analysis of variance (PERMANOVA) using the adonis2 function in the vegan package with 1,000 permutations. Multivariate Association with Linear Models (MaAsLin2) was used to compare the relative abundances of species between the DMN and control groups, after adjusting for age (58), sex (59), BMI (60), and eGFR (50), that may modulate the gut metagenomes (61). We performed analyses on species with at least a 0.1% relative abundance. Differences were considered statistically significant when the false discovery rate (FDR)-corrected *q* value was <0.25. MaAsLin2 was also used to identify significant KEGG gene families between the DMN and control groups using the same adjustment of variables. To compare stool metabolites between groups, multivariate statistical analyses were performed using SIMCA-P version 11.0 (Umetrics, Umeå, Sweden) and OriginPro 8 (OriginLab Corporation, Northampton, MA, USA). Spectral binning data were normalized using mean centering and Pareto scaling. Orthogonal projections to latent structure discriminant analysis (OPLS-DA) was used to discriminate between the DMN and control groups. The peaks assigned to a specific metabolite were compared by a *t* test between the DMN and control groups. We then ran random-forest models to identify which features of the clinical characteristics, stool microbiota, related gene families, and stool metabolites might predict DMN. As predictive features in random-forest models, clinical characteristics (including age, sex, BMI, urine protein-to-creatinine ratio [UPCR], and eGFR), the relative abundances of bacterial species and gene families (related to carbohydrate, protein, and lipid metabolism, which were significant in MaAsLin2 multivariable analyses), and the normalized abundance of significantly different metabolites in the NMR study were included. Additionally, by leveraging random-forest models, we analyzed receiver operating characteristic curves and *C* statistics to predict DMN. For training, testing, and validating the models, 70%, 30%, and 100% of the data were used, respectively. For the evaluation of specific metabolic pathways, the differences in specific gene families associated with methionine and branched-chain amino acids (BCAAs), which were significant by MaAsLin2 analysis, were compared again using the Kruskal-Wallis test. Statistical analyses were performed using R version 4.0.3 (R Core Team) and Python 3.7 (Python Software Foundation, Fredericksburg, VA, USA).

### Data availability.

The sequencing data obtained from this study are available through the European Nucleotide Archive (ENA) at the European Bioinformatics Institute (EBI) under accession number PRJEB57320, and the metabolomic data are available through MetaboLights (http://www.ebi.ac.uk/metabolights) under study accession number MTBLS6331.
