# Supplementary material for: Gut Microbial Genes and Metabolism for Methionine and Branched-Chain Amino Acids in Diabetic Nephropathy
Source: Microbiol Spectr. 2023 Mar 6;11(2):e02344-22. doi: 10.1128/spectrum.02344-22 (PMC10100834; doi:10.1128/spectrum.02344-22)

Supplementary table 1. Significantly different 216 gene families from gut metagenomes in diabetic nephropathy compared to healthy controls.

Gene famliy	Group	coefficient	standard error	p-value	q-value	KEGG hierarchy A	KEGG hierarchy B	KEGG hierarchy C	Description
K07248	DMN	2.13E-05	5.95E-06	0.0070436	0.17176509	ko09100	ko09101	ko00620	aldA; lactaldehyde dehydrogenase / glycolaldehyde dehydrogenase [EC:1.2.1.22 1.2.1.21]
K01712	DMN	2.45E-05	8.94E-06	0.0062338	0.15563785	ko09100	ko09105	ko00340	hutU, UROC1; urocanate hydratase [EC:4.2.1.49]
K03781	DMN	2.46E-05	3.65E-06	0.0075603	0.18266659	ko09100	ko09101	ko00630	katE, CAT, catB, srpA; catalase [EC:1.11.1.6]
K00239	DMN	2.47E-05	8.74E-06	0.0086201	0.20131574	ko09100	ko09101	ko00020	sdhA, frdA; succinate dehydrogenase / fumarate reductase, flavoprotein subunit [EC:1.3.5.1 1.3.5.4]
K08325	DMN	2.52E-05	8.13E-06	0.0022073	0.06393721	ko09100	ko09101	ko00640	yqhD; NADP-dependent alcohol dehydrogenase [EC:1.1.-.-]
K01176	DMN	2.55E-05	7.44E-06	0.0014656	0.04699698	ko09100	ko09101	ko00500	AMY, amyA, malS; alpha-amylase [EC:3.2.1.1]
K01953	DMN	2.64E-05	1.01E-05	0.0089114	0.20582525	ko09100	ko09105	ko00250	asnB, ASNS; asparagine synthase (glutamine-hydrolysing) [EC:6.3.5.4]
K00343	DMN	2.64E-05	6.69E-06	0.0016171	0.05076825	ko09100	ko09102	ko00190	nuoN; NADH-quinone oxidoreductase subunit N [EC:7.1.1.2]
K17103	DMN	2.69E-05	1.01E-05	0.0076675	0.18398525	ko09100	ko09103	ko00564	CHO1, pssA; CDP-diacylglycerol---serine O-phosphatidyltransferase [EC:2.7.8.8]
K01956	DMN	2.70E-05	1.02E-05	0.0083313	0.19588007	ko09100	ko09104	ko00240	carA, CPA1; carbamoyl-phosphate synthase small subunit [EC:6.3.5.5]
K00341	DMN	2.70E-05	7.73E-06	0.0008254	0.03168653	ko09100	ko09102	ko00190	nuoL; NADH-quinone oxidoreductase subunit L [EC:7.1.1.2]
K00609	DMN	2.72E-05	9.24E-06	0.0033272	0.0903867	ko09100	ko09104	ko00240	pyrB, PYR2; aspartate carbamoyltransferase catalytic subunit [EC:2.1.3.2]
K00919	DMN	2.82E-05	9.69E-06	0.0035901	0.09677581	ko09100	ko09109	ko00900	ispE; 4-diphosphocytidyl-2-C-methyl-D-erythritol kinase [EC:2.7.1.148]
K00259	DMN	2.83E-05	7.88E-06	0.0004687	0.02264889	ko09100	ko09105	ko00250	ald; alanine dehydrogenase [EC:1.4.1.1]
K00858	DMN	2.84E-05	1.06E-05	0.0075267	0.18227526	ko09100	ko09108	ko00760	ppnK, NADK; NAD+ kinase [EC:2.7.1.23]
K00705	DMN	2.85E-05	9.63E-06	0.0031022	0.08582867	ko09100	ko09101	ko00500	malQ; 4-alpha-glucanotransferase [EC:2.4.1.25]
K01465	DMN	2.87E-05	1.09E-05	0.0088041	0.20424741	ko09100	ko09104	ko00240	URA4, pyrC; dihydroorotase [EC:3.5.2.3]
K03644	DMN	2.87E-05	8.62E-06	0.0009667	0.03544491	ko09100	ko09108	ko00785	lipA, LIAS, LIP1, LIP5; lipoyl synthase [EC:2.8.1.8]
K01468	DMN	2.88E-05	8.33E-06	0.0007103	0.02920733	ko09100	ko09105	ko00340	hutI, AMDHD1; imidazolonepropionase [EC:3.5.2.7]
K00925	DMN	2.88E-05	1.04E-05	0.0055974	0.14246231	ko09100	ko09101	ko00620	ackA; acetate kinase [EC:2.7.2.1]
K01939	DMN	2.88E-05	1.08E-05	0.0075984	0.18316599	ko09100	ko09104	ko00230	purA, ADSS; adenylosuccinate synthase [EC:6.3.4.4]
K00029	DMN	2.90E-05	8.63E-06	0.00088	0.03249095	ko09100	ko09101	ko00620	E1.1.1.40, maeB; malate dehydrogenase (oxaloacetate-decarboxylating)(NADP+) [EC:1.1.1.40]
K03801	DMN	2.91E-05	9.25E-06	0.0016947	0.05273182	ko09100	ko09108	ko00785	lipB; lipoyl(octanoyl) transferase [EC:2.3.1.181]
K01000	DMN	2.91E-05	1.06E-05	0.0063136	0.15655804	ko09100	ko09107	ko00550	mraY; phospho-N-acetylmuramoyl-pentapeptide-transferase [EC:2.7.8.13]
K01703	DMN	2.97E-05	1.08E-05	0.0059481	0.14921573	ko09100	ko09101	ko00660	leuC, IPMI-L; 3-isopropylmalate/(R)-2-methylmalate dehydratase large subunit [EC:4.2.1.33 4.2.1.35]
K09458	DMN	2.97E-05	1.07E-05	0.0056544	0.14305657	ko09100	ko09103	ko00061	fabF, OXSM, CEM1; 3-oxoacyl-[acyl-carrier-protein] synthase II [EC:2.3.1.179]
K01895	DMN	2.98E-05	7.98E-06	0.0003101	0.01776918	ko09100	ko09101	ko00010	ACSS1_2, acs; acetyl-CoA synthetase [EC:6.2.1.1]
K00826	DMN	2.99E-05	1.00E-05	0.0028776	0.08089687	ko09100	ko09105	ko00270	E2.6.1.42, ilvE; branched-chain amino acid aminotransferase [EC:2.6.1.42]
K05366	DMN	2.99E-05	7.97E-06	0.0002893	0.01711164	ko09100	ko09107	ko00550	mrcA; penicillin-binding protein 1A [EC:2.4.1.129 3.4.16.4]
K00281	DMN	2.99E-05	7.44E-06	0.0002678	0.01711164	ko09100	ko09101	ko00630	GLDC, gcvP; glycine dehydrogenase [EC:1.4.4.2]
K17828	DMN	3.00E-05	9.39E-06	0.0013946	0.04542658	ko09100	ko09104	ko00240	pyrDI; dihydroorotate dehydrogenase (NAD+) catalytic subunit [EC:1.3.1.14]
K05515	DMN	3.00E-05	9.29E-06	0.0012344	0.04188961	ko09100	ko09107	ko00550	mrdA; penicillin-binding protein 2 [EC:3.4.16.4]
K02536	DMN	3.02E-05	8.09E-06	0.0002895	0.01711164	ko09100	ko09107	ko00540	lpxD; UDP-3-O-[3-hydroxymyristoyl] glucosamine N-acyltransferase [EC:2.3.1.191]
K02548	DMN	3.03E-05	1.02E-05	0.0030528	0.08491026	ko09100	ko09108	ko00130	menA; 1,4-dihydroxy-2-naphthoate polyprenyltransferase [EC:2.5.1.74]
K01885	DMN	3.03E-05	6.76E-06	0.0004993	0.02320642	ko09100	ko09108	ko00860	EARS, gltX; glutamyl-tRNA synthetase [EC:6.1.1.17]
K01843	DMN	3.03E-05	8.72E-06	0.0005433	0.02434524	ko09100	ko09105	ko00310	kamA; lysine 2,3-aminomutase [EC:5.4.3.2]
K00548	DMN	3.04E-05	8.14E-06	0.0002611	0.01711164	ko09100	ko09105	ko00270	metH, MTR; 5-methyltetrahydrofolate--homocysteine methyltransferase [EC:2.1.1.13]
K03737	DMN	3.05E-05	9.08E-06	0.0008113	0.03162555	ko09100	ko09101	ko00010	por, nifJ; pyruvate-ferredoxin/flavodoxin oxidoreductase [EC:1.2.7.1 1.2.7.-]
K00873	DMN	3.06E-05	1.20E-05	0.0113691	0.2499306	ko09100	ko09101	ko00010	PK, pyk; pyruvate kinase [EC:2.7.1.40]
K00757	DMN	3.06E-05	9.97E-06	0.0021272	0.06287513	ko09100	ko09104	ko00240	udp, UPP; uridine phosphorylase [EC:2.4.2.3]
K00145	DMN	3.06E-05	1.06E-05	0.0040641	0.10734596	ko09100	ko09105	ko00220	argC; N-acetyl-gamma-glutamyl-phosphate reductase [EC:1.2.1.38]
K02118	DMN	3.07E-05	9.38E-06	0.0010509	0.03722782	ko09100	ko09102	ko00190	ATPVB, ntpB, atpB; V/A-type H+/Na+-transporting ATPase subunit B
K01079	DMN	3.07E-05	7.01E-06	0.000365	0.01952851	ko09100	ko09102	ko00680	serB, PSPH; phosphoserine phosphatase [EC:3.1.3.3]
K00342	DMN	3.08E-05	8.22E-06	0.0002506	0.01684217	ko09100	ko09102	ko00190	nuoM; NADH-quinone oxidoreductase subunit M [EC:7.1.1.2]
K03787	DMN	3.09E-05	9.33E-06	0.0009713	0.03545349	ko09100	ko09104	ko00230	surE; 5'-nucleotidase [EC:3.1.3.5]
K06001	DMN	3.09E-05	9.52E-06	0.0011569	0.03991197	ko09100	ko09105	ko00260	trpB; tryptophan synthase beta chain [EC:4.2.1.20]
K16363	DMN	3.10E-05	8.78E-06	0.0004316	0.0215979	ko09100	ko09103	ko00061	lpxC-fabZ; UDP-3-O-[3-hydroxymyristoyl] N-acetylglucosamine deacetylase / 3-hydroxyacyl-[acyl-carrier-protein] dehydratase [EC:3.5.1.108 4.2.1.59]
K01089	DMN	3.11E-05	8.56E-06	0.0003187	0.0181493	ko09100	ko09105	ko00340	hisB; imidazoleglycerol-phosphate dehydratase / histidinol-phosphatase [EC:4.2.1.19 3.1.3.15]
K01082	DMN	3.12E-05	9.50E-06	0.0010358	0.0371766	ko09100	ko09102	ko00920	cysQ, MET22, BPNT1; 3'(2'), 5'-bisphosphate nucleotidase [EC:3.1.3.7]
K01945	DMN	3.12E-05	1.03E-05	0.0024568	0.07038928	ko09100	ko09104	ko00230	purD; phosphoribosylamine---glycine ligase [EC:6.3.4.13]
K01937	DMN	3.12E-05	8.66E-06	0.0003437	0.01887057	ko09100	ko09104	ko00240	pyrG, CTPS; CTP synthase [EC:6.3.4.2]
K00018	DMN	3.12E-05	9.31E-06	0.0008211	0.03168653	ko09100	ko09101	ko00630	hprA; glycerate dehydrogenase [EC:1.1.1.29]
K02123	DMN	3.12E-05	7.72E-06	0.0001334	0.01271591	ko09100	ko09102	ko00190	ATPVI, ntpI, atpI; V/A-type H+/Na+-transporting ATPase subunit I
K13378	DMN	3.12E-05	8.06E-06	0.0001754	0.01403683	ko09100	ko09102	ko00190	nuoCD; NADH-quinone oxidoreductase subunit C/D [EC:7.1.1.2]
K01676	DMN	3.12E-05	1.01E-05	0.001933	0.0586302	ko09100	ko09101	ko00020	E4.2.1.2A, fumA, fumB; fumarate hydratase, class I [EC:4.2.1.2]
K00605	DMN	3.13E-05	9.64E-06	0.00119	0.04091424	ko09100	ko09101	ko00630	gcvT, AMT; aminomethyltransferase [EC:2.1.2.10]
K12343	DMN	3.13E-05	9.26E-06	0.0007492	0.03000502	ko09100	ko09103	ko00140	SRD5A1; 3-oxo-5-alpha-steroid 4-dehydrogenase 1 [EC:1.3.1.22]
K01735	DMN	3.15E-05	9.59E-06	0.0010423	0.0371766	ko09100	ko09105	ko00400	aroB; 3-dehydroquinate synthase [EC:4.2.3.4]
K01689	DMN	3.15E-05	1.19E-05	0.0086048	0.20131574	ko09100	ko09101	ko00010	ENO, eno; enolase [EC:4.2.1.11]
K00337	DMN	3.15E-05	8.18E-06	0.0001726	0.01402891	ko09100	ko09102	ko00190	nuoH; NADH-quinone oxidoreductase subunit H [EC:7.1.1.2]
K01803	DMN	3.16E-05	1.21E-05	0.0092481	0.21266636	ko09100	ko09101	ko00010	TPI, tpiA; triosephosphate isomerase (TIM) [EC:5.3.1.1]
K01491	DMN	3.16E-05	1.13E-05	0.0054117	0.13874572	ko09100	ko09102	ko00720	folD; methylenetetrahydrofolate dehydrogenase (NADP+) / methenyltetrahydrofolate cyclohydrolase [EC:1.5.1.5 3.5.4.9]
K01804	DMN	3.17E-05	8.32E-06	0.0001865	0.01459214	ko09100	ko09101	ko00040	araA; L-arabinose isomerase [EC:5.3.1.4]
K01952	DMN	3.17E-05	8.87E-06	0.0050293	0.13053718	ko09100	ko09104	ko00230	PFAS, purL; phosphoribosylformylglycinamidine synthase [EC:6.3.5.3]
K00703	DMN	3.18E-05	1.14E-05	0.0056617	0.14305657	ko09100	ko09101	ko00500	glgA; starch synthase [EC:2.4.1.21]
K00948	DMN	3.18E-05	1.04E-05	0.0021515	0.06287513	ko09100	ko09101	ko00030	PRPS, prsA; ribose-phosphate pyrophosphokinase [EC:2.7.6.1]

K13038	DMN	3.19E-05	8.16E-06	0.0001591	0.0137888	ko09100	ko09108	ko00770	coaBC, dfp; phosphopantothenoylcysteine decarboxylase / phosphopantothenate---cysteine ligase [EC:4.1.1.36 6.3.2.5]
K02523	DMN	3.19E-05	9.23E-06	0.0005533	0.02468797	ko09100	ko09109	ko00900	ispB; octaprenyl-diphosphate synthase [EC:2.5.1.90]
K00912	DMN	3.21E-05	9.05E-06	0.0004136	0.02136582	ko09100	ko09107	ko00540	lpxK; tetraacyldisaccharide 4'-kinase [EC:2.7.1.130]
K00927	DMN	3.21E-05	9.42E-06	0.0006658	0.02815092	ko09100	ko09101	ko00010	PGK, pgk; phosphoglycerate kinase [EC:2.7.2.3]
K09011	DMN	3.21E-05	8.42E-06	0.0001744	0.01403683	ko09100	ko09101	ko00660	cimA; (R)-citramalate synthase [EC:2.3.1.182]
K08289	DMN	3.23E-05	7.53E-06	0.000106	0.01078749	ko09100	ko09104	ko00230	purT; phosphoribosylglycinamide formyltransferase 2 [EC:2.1.2.2]
K03474	DMN	3.23E-05	1.02E-05	0.0015877	0.05014756	ko09100	ko09108	ko00750	pdxJ; pyridoxine 5-phosphate synthase [EC:2.6.99.2]
K01719	DMN	3.25E-05	7.22E-06	0.0001531	0.01368345	ko09100	ko09108	ko00860	hemD, UROS; uroporphyrinogen-III synthase [EC:4.2.1.75]
K01810	DMN	3.25E-05	1.02E-05	0.0014538	0.04676279	ko09100	ko09101	ko00010	GPI, pgi; glucose-6-phosphate isomerase [EC:5.3.1.9]
K00133	DMN	3.26E-05	1.06E-05	0.0020215	0.06108666	ko09100	ko09105	ko00260	asd; aspartate-semialdehyde dehydrogenase [EC:1.2.1.11]
K00226	DMN	3.28E-05	8.83E-06	0.0002203	0.01615782	ko09100	ko09104	ko00240	pyrD; dihydroorotate dehydrogenase (fumarate) [EC:1.3.98.1]
K00278	DMN	3.29E-05	9.18E-06	0.0003427	0.01887057	ko09100	ko09105	ko00250	nadB; L-aspartate oxidase [EC:1.4.3.16]
K13789	DMN	3.29E-05	9.35E-06	0.0004315	0.0215979	ko09100	ko09109	ko00900	GGPS; geranylgeranyl diphosphate synthase, type II [EC:2.5.1.1 2.5.1.10 2.5.1.29]
K00075	DMN	3.30E-05	1.05E-05	0.001688	0.05273182	ko09100	ko09101	ko00520	murB; UDP-N-acetylmuramate dehydrogenase [EC:1.3.1.98]
K00931	DMN	3.33E-05	1.08E-05	0.0021399	0.06287513	ko09100	ko09105	ko00330	proB; glutamate 5-kinase [EC:2.7.2.11]
K00208	DMN	3.33E-05	8.64E-06	0.0001367	0.01291439	ko09100	ko09103	ko00061	fabI; enoyl-[acyl-carrier protein] reductase I [EC:1.3.1.9 1.3.1.10]
K01928	DMN	3.34E-05	9.72E-06	0.0005822	0.0255667	ko09100	ko09105	ko00300	murE; UDP-N-acetylmuramoyl-L-alanyl-D-glutamate--2,6-diaminopimelate ligase [EC:6.3.2.13]
K01438	DMN	3.34E-05	9.50E-06	0.0004367	0.0215979	ko09100	ko09105	ko00220	argE; acetylornithine deacetylase [EC:3.5.1.16]
K00645	DMN	3.35E-05	9.90E-06	0.0007049	0.02910181	ko09100	ko09103	ko00061	fabD, MCAT, MCT1; [acyl-carrier-protein] S-malonyltransferase [EC:2.3.1.39]
K00762	DMN	3.36E-05	1.30E-05	0.010333	0.23201612	ko09100	ko09104	ko00240	pyrE; orotate phosphoribosyltransferase [EC:2.4.2.10]
K07405	DMN	3.36E-05	8.43E-06	7.58E-05	0.01017507	ko09100	ko09101	ko00500	E3.2.1.1A; alpha-amylase [EC:3.2.1.1]
K02117	DMN	3.37E-05	8.72E-06	0.0001258	0.0122156	ko09100	ko09102	ko00190	ATPVA, ntpA, atpA; V/A-type H+/Na+-transporting ATPase subunit A [EC:7.1.2.2 7.2.2.1]
K01580	DMN	3.37E-05	9.23E-06	0.000267	0.01711164	ko09100	ko09101	ko00650	E4.1.1.15, gadB, gadA, GAD; glutamate decarboxylase [EC:4.1.1.15]
K02500	DMN	3.38E-05	9.75E-06	0.0005322	0.02434284	ko09100	ko09105	ko00340	hisF; imidazole glycerol-phosphate synthase subunit HisF [EC:4.3.2.10]
K00057	DMN	3.38E-05	9.42E-06	0.0003385	0.01877774	ko09100	ko09103	ko00564	gpsA; glycerol-3-phosphate dehydrogenase (NAD(P)+) [EC:1.1.1.94]
K00147	DMN	3.39E-05	1.02E-05	0.0008762	0.03246771	ko09100	ko09105	ko00330	proA; glutamate-5-semialdehyde dehydrogenase [EC:1.2.1.41]
K00929	DMN	3.39E-05	9.94E-06	0.000644	0.02748667	ko09100	ko09101	ko00650	buk; butyrate kinase [EC:2.7.2.7]
K01756	DMN	3.39E-05	1.02E-05	0.0008726	0.03246771	ko09100	ko09104	ko00230	purB, ADSL; adenylosuccinate lyase [EC:4.3.2.2]
K01798	DMN	3.40E-05	8.25E-06	5.53E-05	0.0095013	ko09100	ko09105	ko00300	alr-murF; Alr-MurF fusion protein [EC:5.1.1.1 6.3.2.10]
K11754	DMN	3.40E-05	8.96E-06	0.0001559	0.01368345	ko09100	ko09108	ko00790	folC; dihydrofolate synthase / folylpolyglutamate synthase [EC:6.3.2.12 6.3.2.17]
K00600	DMN	3.40E-05	9.01E-06	0.0001707	0.01398737	ko09100	ko09101	ko00630	glyA, SHMT; glycine hydroxymethyltransferase [EC:2.1.2.1]
K03778	DMN	3.41E-05	8.31E-06	5.87E-05	0.00963694	ko09100	ko09101	ko00620	ldhA; D-lactate dehydrogenase [EC:1.1.1.28]
K06133	DMN	3.42E-05	1.04E-05	0.0010375	0.0371766	ko09100	ko09108	ko00770	LYS5, acpT; 4'-phosphopantetheinyl transferase [EC:2.7.8.-]
K00748	DMN	3.43E-05	8.57E-06	8.34E-05	0.01017507	ko09100	ko09107	ko00540	lpxB; lipid-A-disaccharide synthase [EC:2.4.1.182]
K07106	DMN	3.43E-05	9.80E-06	0.0004579	0.02223128	ko09100	ko09101	ko00520	murQ; N-acetylmuramic acid 6-phosphate etherase [EC:4.2.1.126]
K11717	DMN	3.44E-05	8.83E-06	0.0001133	0.01131536	ko09100	ko09106	ko00450	sufS; cysteine desulfurase / selenocysteine lyase [EC:2.8.1.7 4.4.1.16]
K01921	DMN	3.44E-05	1.10E-05	0.0018485	0.05667628	ko09100	ko09106	ko00473	ddl; D-alanine-D-alanine ligase [EC:6.3.2.4]
K03269	DMN	3.44E-05	9.88E-06	0.0005002	0.02320642	ko09100	ko09107	ko00540	lpxH; UDP-2,3-diacylglucosamine hydrolase [EC:3.6.1.54]
K00790	DMN	3.45E-05	1.06E-05	0.0011004	0.03859291	ko09100	ko09101	ko00520	murA; UDP-N-acetylglucosamine 1-carboxyvinyltransferase [EC:2.5.1.7]
K01755	DMN	3.45E-05	1.00E-05	0.0005758	0.02547445	ko09100	ko09105	ko00250	argH, ASL; argininosuccinate lyase [EC:4.3.2.1]
K00765	DMN	3.47E-05	9.12E-06	0.0001503	0.01368345	ko09100	ko09105	ko00340	hisG; ATP phosphoribosyltransferase [EC:2.4.2.17]
K01933	DMN	3.47E-05	9.56E-06	0.0002824	0.01711164	ko09100	ko09104	ko00230	purM; phosphoribosylformylglycinamidine cyclo-ligase [EC:6.3.3.1]
K00831	DMN	3.49E-05	8.10E-06	3.32E-05	0.00772891	ko09100	ko09102	ko00680	serC, PSAT1; phosphoserine aminotransferase [EC:2.6.1.52]
K01585	DMN	3.49E-05	1.02E-05	0.0006424	0.02748667	ko09100	ko09105	ko00330	speA; arginine decarboxylase [EC:4.1.1.19]
K13566	DMN	3.50E-05	7.91E-06	3.99E-05	0.00784687	ko09100	ko09105	ko00250	NIT2, yafV; omega-amidase [EC:3.5.1.3]
K01778	DMN	3.51E-05	1.05E-05	0.0008409	0.03205011	ko09100	ko09105	ko00300	dapF; diaminopimelate epimerase [EC:5.1.1.7]
K02517	DMN	3.51E-05	9.65E-06	0.0002769	0.01711164	ko09100	ko09107	ko00540	lpxL, htrB; Kdo2-lipid IVA lauroyltransferase/acyltransferase [EC:2.3.1.241 2.3.1.-]
K00099	DMN	3.51E-05	9.26E-06	0.0001544	0.01368345	ko09100	ko09109	ko00900	dxr; 1-deoxy-D-xylulose-5-phosphate reductoisomerase [EC:1.1.1.267]
K02501	DMN	3.52E-05	1.17E-05	0.0026859	0.07653475	ko09100	ko09105	ko00340	hisH; imidazole glycerol-phosphate synthase subunit HisH [EC:4.3.2.10]
K00767	DMN	3.53E-05	8.93E-06	8.80E-05	0.01017507	ko09100	ko09108	ko00760	nadC, QPRT; nicotinate-nucleotide pyrophosphorylase (carboxylating) [EC:2.4.2.19]
K06167	DMN	3.53E-05	1.01E-05	0.0004885	0.0230031	ko09100	ko09106	ko00440	phnP; phosphoribosyl 1,2-cyclic phosphate phosphodiesterase [EC:3.1.4.55]
K00793	DMN	3.54E-05	1.02E-05	0.0005399	0.02434284	ko09100	ko09108	ko00740	ribE, RIB5; riboflavin synthase [EC:2.5.1.9]
K00052	DMN	3.54E-05	1.02E-05	0.0005409	0.02434284	ko09100	ko09101	ko00660	leuB, IMDH; 3-isopropylmalate dehydrogenase [EC:1.1.1.85]
K02527	DMN	3.54E-05	8.74E-06	6.16E-05	0.00963694	ko09100	ko09107	ko00540	kdtA, waaA; 3-deoxy-D-manno-octulosonic-acid transferase [EC:2.4.99.12 2.4.99.13 2.4.99.14 2.4.99.15]
K01736	DMN	3.55E-05	9.38E-06	0.0001548	0.01368345	ko09100	ko09105	ko00400	aroC; chorismate synthase [EC:4.2.3.5]
K01687	DMN	3.55E-05	1.00E-05	0.0004066	0.02110446	ko09100	ko09105	ko00290	ilvD; dihydroxy-acid dehydratase [EC:4.2.1.9]
K04518	DMN	3.56E-05	1.02E-05	0.0004892	0.0230031	ko09100	ko09105	ko00400	pheA2; prephenate dehydratase [EC:4.2.1.51]
K03473	DMN	3.56E-05	9.46E-06	0.0001678	0.01398737	ko09100	ko09108	ko00750	pdxB; erythronate-4-phosphate dehydrogenase [EC:1.1.1.290]
K15633	DMN	3.57E-05	9.75E-06	0.000246	0.01679208	ko09100	ko09101	ko00010	gpmI; 2,3-bisphosphoglycerate-independent phosphoglycerate mutase [EC:5.4.2.12]
K00088	DMN	3.60E-05	9.69E-06	0.000208	0.01569062	ko09100	ko09104	ko00230	IMPDH, guaB; IMP dehydrogenase [EC:1.1.1.205]
K00849	DMN	3.60E-05	9.52E-06	0.0001566	0.01368345	ko09100	ko09101	ko00052	galK; galactokinase [EC:2.7.1.6]
K11753	DMN	3.61E-05	1.04E-05	0.000515	0.02378832	ko09100	ko09108	ko00740	ribF; riboflavin kinase / FMN adenylyltransferase [EC:2.7.1.26 2.7.7.2]
K03783	DMN	3.62E-05	8.63E-06	3.28E-05	0.00772891	ko09100	ko09104	ko00230	punA, PNP; purine-nucleoside phosphorylase [EC:2.4.2.1]
K00331	DMN	3.63E-05	1.12E-05	0.0012088	0.04128908	ko09100	ko09102	ko00190	nuoB; NADH-quinone oxidoreductase subunit B [EC:7.1.1.2]
K01929	DMN	3.67E-05	9.36E-06	9.11E-05	0.01017507	ko09100	ko09105	ko00300	murF; UDP-N-acetylmuramoyl-tripeptide--D-alanyl-D-alanine ligase [EC:6.3.2.10]
K01649	DMN	3.68E-05	8.87E-06	4.08E-05	0.00784687	ko09100	ko09101	ko00620	leuA, IMS; 2-isopropylmalate synthase [EC:2.3.3.13]
K06041	DMN	3.69E-05	1.19E-05	0.0021035	0.06266181	ko09100	ko09107	ko00540	kdsD, kpsF; arabinose-5-phosphate isomerase [EC:5.3.1.13]
K00606	DMN	3.70E-05	9.25E-06	6.79E-05	0.00993039	ko09100	ko09108	ko00770	panB; 3-methyl-2-oxobutanoate hydroxymethyltransferase [EC:2.1.2.11]
K00789	DMN	3.73E-05	1.14E-05	0.0011493	0.03990502	ko09100	ko09105	ko00270	metK; S-adenosylmethionine synthetase [EC:2.5.1.6]
K00013	DMN	3.74E-05	8.88E-06	3.02E-05	0.00766879	ko09100	ko09105	ko00340	hisD; histidinol dehydrogenase [EC:1.1.1.23]
K00215	DMN	3.75E-05	1.13E-05	0.0009173	0.03375128	ko09100	ko09105	ko00300	dapB; 4-hydroxy-tetrahydrodipicolinate reductase [EC:1.17.1.8]

K00857	DMN	3.76E-05	1.05E-05	0.000361	0.01941248	ko09100	ko09104	ko00240	tdk, TK; thymidine kinase [EC:2.7.1.21]
K01071	DMN	3.76E-05	8.42E-06	9.84E-06	0.00396781	ko09100	ko09103	ko00061	MCH; medium-chain acyl-[acyl-carrier-protein] hydrolase [EC:3.1.2.21]
K03517	DMN	3.77E-05	1.00E-05	0.0001664	0.01398737	ko09100	ko09108	ko00760	nadA; quinolinate synthase [EC:2.5.1.72]
K00012	DMN	3.78E-05	1.03E-05	0.0002375	0.01666423	ko09100	ko09101	ko00040	UGDH, ugd; UDPglucose 6-dehydrogenase [EC:1.1.1.22]
K01840	DMN	3.82E-05	8.00E-06	6.41E-06	0.00305378	ko09100	ko09101	ko00051	manB; phosphomannomutase [EC:5.4.2.8]
K09903	DMN	3.84E-05	1.08E-05	0.0003931	0.02061284	ko09100	ko09104	ko00240	pyrH; uridylate kinase [EC:2.7.4.22]
K00979	DMN	3.84E-05	9.30E-06	3.80E-05	0.00784687	ko09100	ko09107	ko00540	kdsB; 3-deoxy-manno-octulosonate cytidylyltransferase (CMP-KDO synthetase) [EC:2.7.7.38]
K01925	DMN	3.85E-05	1.05E-05	0.0002482	0.01679208	ko09100	ko09106	ko00471	murD; UDP-N-acetylmuramoylalanine--D-glutamate ligase [EC:6.3.2.9]
K01619	DMN	3.85E-05	1.35E-05	0.0046669	0.12213912	ko09100	ko09101	ko00030	deoC, DERA; deoxyribose-phosphate aldolase [EC:4.1.2.4]
K01591	DMN	3.87E-05	9.55E-06	5.09E-05	0.00890259	ko09100	ko09104	ko00240	pyrF; orotidine-5'-phosphate decarboxylase [EC:4.1.1.23]
K10026	DMN	3.89E-05	1.16E-05	0.0008499	0.03217274	ko09100	ko09108	ko00790	queE; 7-carboxy-7-deazaguanine synthase [EC:4.3.99.3]
K01745	DMN	3.89E-05	9.27E-06	2.74E-05	0.00750032	ko09100	ko09105	ko00340	hutH, HAL; histidine ammonia-lyase [EC:4.3.1.3]
K03527	DMN	3.90E-05	9.42E-06	3.63E-05	0.00784687	ko09100	ko09109	ko00900	ispH, lytB; 4-hydroxy-3-methylbut-2-en-1-yl diphosphate reductase [EC:1.17.7.4]
K03183	DMN	3.93E-05	9.13E-06	1.86E-05	0.00649201	ko09100	ko09108	ko00130	ubiE; demethylmenaquinone methyltransferase / 2-methoxy-6-polyprenyl-1,4-benzoquinol methylase [EC:2.1.1.163 2.1.1.201]
K02121	DMN	3.95E-05	1.09E-05	0.0003059	0.01772156	ko09100	ko09102	ko00190	ATPVE, ntpE, atpE; V/A-type H+/Na+-transporting ATPase subunit E
K00796	DMN	3.99E-05	1.01E-05	8.42E-05	0.01017507	ko09100	ko09108	ko00790	folP; dihydropteroate synthase [EC:2.5.1.15]
K00588	DMN	4.05E-05	1.07E-05	0.0001641	0.01398737	ko09100	ko09110	ko00940	E2.1.1.104; caffeoyl-CoA O-methyltransferase [EC:2.1.1.104]
K01923	DMN	4.06E-05	1.02E-05	6.68E-05	0.00993039	ko09100	ko09104	ko00230	purC; phosphoribosylaminoimidazole-succinocarboxamide synthase [EC:6.3.2.6]
K06153	DMN	4.06E-05	1.10E-05	0.0002274	0.01656207	ko09100	ko09107	ko00550	bacA; undecaprenyl-diphosphatase [EC:3.6.1.27]
K01814	DMN	4.12E-05	1.04E-05	7.21E-05	0.01008276	ko09100	ko09105	ko00340	hisA; phosphoribosylformimino-5-aminoimidazole carboxamide ribotide isomerase [EC:5.3.1.16]
K00859	DMN	4.12E-05	1.11E-05	0.0002022	0.01536357	ko09100	ko09108	ko00770	coaE; dephospho-CoA kinase [EC:2.7.1.24]
K00991	DMN	4.13E-05	9.60E-06	1.75E-05	0.00631762	ko09100	ko09109	ko00900	ispD; 2-C-methyl-D-erythritol 4-phosphate cytidylyltransferase [EC:2.7.7.60]
K00339	DMN	4.13E-05	1.29E-05	0.0015007	0.0478324	ko09100	ko09102	ko00190	nuoJ; NADH-quinone oxidoreductase subunit J [EC:7.1.1.2]
K02120	DMN	4.15E-05	1.12E-05	0.000214	0.01591632	ko09100	ko09102	ko00190	ATPVD, ntpD, atpD; V/A-type H+/Na+-transporting ATPase subunit D
K01627	DMN	4.18E-05	8.51E-06	1.95E-06	0.00145742	ko09100	ko09107	ko00540	kdsA; 2-dehydro-3-deoxyphosphooctonate aldolase (KDO 8-P synthase) [EC:2.5.1.55]
K01613	DMN	4.19E-05	9.87E-06	2.18E-05	0.00714119	ko09100	ko09103	ko00564	psd, PISD; phosphatidylserine decarboxylase [EC:4.1.1.65]
K01209	DMN	4.23E-05	1.34E-05	0.001761	0.05422609	ko09100	ko09101	ko00520	abfA; alpha-L-arabinofuranosidase [EC:3.2.1.55]
K03524	DMN	4.26E-05	1.04E-05	3.96E-05	0.00784687	ko09100	ko09108	ko00780	birA; BirA family transcriptional regulator, biotin operon repressor / biotin---[acetyl-CoA-carboxylase] ligase [EC:6.3.4.15]
K01960	DMN	4.28E-05	1.43E-05	0.0080117	0.19093344	ko09100	ko09101	ko00020	pycB; pyruvate carboxylase subunit B [EC:6.4.1.1]
K12410	DMN	4.29E-05	1.05E-05	4.19E-05	0.00784687	ko09100	ko09108	ko00760	npdA; NAD-dependent deacetylase [EC:2.3.1.286]
K00930	DMN	4.31E-05	1.03E-05	2.79E-05	0.00750032	ko09100	ko09105	ko00220	argB; acetylglutamate kinase [EC:2.7.2.8]
K00616	DMN	4.31E-05	1.10E-05	9.45E-05	0.01025913	ko09100	ko09101	ko00030	E2.2.1.2, talA, talB; transaldolase [EC:2.2.1.2]
K00657	DMN	4.32E-05	1.30E-05	0.0009765	0.03545349	ko09100	ko09105	ko00330	speG, SAT; diamine N-acetyltransferase [EC:2.3.1.57]
K03270	DMN	4.37E-05	1.20E-05	0.0003054	0.01772156	ko09100	ko09107	ko00540	kdsC; 3-deoxy-D-manno-octulosonate 8-phosphate phosphatase (KDO 8-P phosphatase) [EC:3.1.3.45]
K20866	DMN	4.45E-05	1.14E-05	9.66E-05	0.01025913	ko09100	ko09101	ko00010	yihX; glucose-1-phosphatase [EC:3.1.3.10]
K01918	DMN	4.49E-05	8.36E-06	3.62E-07	0.00045935	ko09100	ko09106	ko00410	panC; pantoate--beta-alanine ligase [EC:6.3.2.1]
K00817	DMN	4.52E-05	1.30E-05	0.0005592	0.02484594	ko09100	ko09105	ko00340	hisC; histidinol-phosphate aminotransferase [EC:2.6.1.9]
K01950	DMN	4.56E-05	1.01E-05	6.01E-06	0.00300302	ko09100	ko09108	ko00760	E6.3.5.1, NADSYN1, QNS1, nadE; NAD+ synthase (glutamine-hydrolysing) [EC:6.3.5.1]
K00648	DMN	4.57E-05	1.33E-05	0.0006724	0.02831678	ko09100	ko09103	ko00061	fabH; 3-oxoacyl-[acyl-carrier-protein] synthase III [EC:2.3.1.180]
K01425	DMN	4.65E-05	1.28E-05	0.0003461	0.01890151	ko09100	ko09105	ko00250	glsA, GLS; glutaminase [EC:3.5.1.2]
K21636	DMN	4.65E-05	1.32E-05	0.0004874	0.0230031	ko09100	ko09104	ko00230	nrdD; ribonucleoside-triphosphate reductase (formate) [EC:1.1.98.6]
K08678	DMN	4.70E-05	1.01E-05	3.56E-06	0.00221285	ko09100	ko09101	ko00520	UXS1, uxs; UDP-glucuronate decarboxylase [EC:4.1.1.35]
K01783	DMN	4.75E-05	1.29E-05	0.0002758	0.01711164	ko09100	ko09101	ko00030	rpe, RPE; ribulose-phosphate 3-epimerase [EC:5.1.3.1]
K00602	DMN	4.77E-05	1.65E-05	0.0102531	0.23071701	ko09100	ko09104	ko00230	purH; phosphoribosylaminoimidazolecarboxamide formyltransferase / IMP cyclohydrolase [EC:2.1.2.3 3.5.4.10]
K00639	DMN	4.82E-05	9.59E-06	5.15E-07	0.00054029	ko09100	ko09105	ko00260	kbl, GCAT; glycine C-acetyltransferase [EC:2.3.1.29]
K00297	DMN	4.88E-05	1.18E-05	4.15E-05	0.00784687	ko09100	ko09102	ko00720	metF, MTHFR; methylenetetrahydrofolate reductase (NADPH) [EC:1.5.1.20]
K00939	DMN	4.88E-05	1.23E-05	8.06E-05	0.01017507	ko09100	ko09104	ko00230	adk, AK; adenylate kinase [EC:2.7.4.3]
K00946	DMN	5.00E-05	8.19E-06	1.35E-08	4.70E-05	ko09100	ko09108	ko00730	thiL; thiamine-monophosphate kinase [EC:2.7.4.16]
K00791	DMN	5.04E-05	1.71E-05	0.0088415	0.20466176	ko09100	ko09109	ko00908	miaA, TRIT1; tRNA dimethylallyltransferase [EC:2.5.1.75]
K00928	DMN	5.08E-05	1.66E-05	0.0066956	0.16404289	ko09100	ko09105	ko00260	lysC; aspartate kinase [EC:2.7.2.4]
K00812	DMN	5.09E-05	1.80E-05	0.0112825	0.24915654	ko09100	ko09105	ko00250	aspB; aspartate aminotransferase [EC:2.6.1.1]
K01710	DMN	5.23E-05	1.13E-05	3.85E-06	0.00221285	ko09100	ko09107	ko00541	rfbB, rmlB, rffG; dTDP-glucose 4,6-dehydratase [EC:4.2.1.46]
K06131	DMN	5.24E-05	1.80E-05	0.0081986	0.19450228	ko09100	ko09103	ko00564	clsA_B; cardiolipin synthase A/B [EC:2.7.8.-]
K00973	DMN	5.25E-05	1.32E-05	8.47E-05	0.01017507	ko09100	ko09107	ko00541	rfbA, rmlA, rffH; glucose-1-phosphate thymidylyltransferase [EC:2.7.7.24]
K00821	DMN	5.38E-05	9.90E-06	5.46E-08	0.0001431	ko09100	ko09105	ko00300	argD; acetylornithine/N-succinyldiaminopimelate aminotransferase [EC:2.6.1.11 2.6.1.17]
K01077	DMN	5.38E-05	1.16E-05	4.01E-06	0.00221285	ko09100	ko09108	ko00730	E3.1.3.1, phoA, phoB; alkaline phosphatase [EC:3.1.3.1]
K01424	DMN	5.61E-05	1.95E-05	0.0109622	0.24457307	ko09100	ko09105	ko00250	E3.5.1.1, ansA, ansB; L-asparaginase [EC:3.5.1.1]
K02428	DMN	5.80E-05	1.91E-05	0.0075085	0.18225548	ko09100	ko09104	ko00230	rdgB; XTP/dITP diphosphohydrolase [EC:3.6.1.66]
K00067	DMN	5.91E-05	1.50E-05	0.0011571	0.03991197	ko09100	ko09107	ko00541	rfbD, rmlD; dTDP-4-dehydrorhamnose reductase [EC:1.1.1.133]
K01270	DMN	5.91E-05	1.72E-05	0.0027659	0.07796471	ko09100	ko09106	ko00480	pepD; dipeptidase D [EC:3.4.13.-]
K00262	DMN	5.92E-05	1.63E-05	0.0021254	0.06287513	ko09100	ko09102	ko00910	E1.4.1.4, gdhA; glutamate dehydrogenase (NADP+) [EC:1.4.1.4]
K00024	DMN	5.97E-05	1.66E-05	0.0020916	0.06255978	ko09100	ko09101	ko00020	mdh; malate dehydrogenase [EC:1.1.1.37]
K00876	DMN	6.38E-05	1.93E-05	0.003744	0.0998977	ko09100	ko09104	ko00240	udk, UCK; uridine kinase [EC:2.7.1.48]
K07407	DMN	6.54E-05	1.89E-05	0.0029617	0.08303856	ko09100	ko09101	ko00052	E3.2.1.22B, galA, rafA; alpha-galactosidase [EC:3.2.1.22]
K01847	DMN	6.56E-05	1.71E-05	0.0012064	0.04128908	ko09100	ko09101	ko00630	MUT; methylmalonyl-CoA mutase [EC:5.4.99.2]
K01139	DMN	6.61E-05	1.54E-05	0.0005367	0.02434284	ko09100	ko09104	ko00230	spoT; GTP diphosphokinase / guanosine-3',5'-bis(diphosphate) 3'-diphosphatase [EC:2.7.6.5 3.1.7.2]
K00174	DMN	7.19E-05	1.75E-05	0.000777	0.03062875	ko09100	ko09101	ko00010	korA, oorA, oforA; 2-oxoglutarate/2-oxoacid ferredoxin oxidoreductase subunit alpha [EC:1.2.7.3 1.2.7.11]
K02564	DMN	7.43E-05	2.15E-05	0.0031103	0.08582867	ko09100	ko09101	ko00520	nagB, GNPDA; glucosamine-6-phosphate deaminase [EC:3.5.99.6]

K01790	DMN	7.85E-05	1.86E-05	0.0006257	0.02699899	ko09100	ko09107	ko00541	rfbC, rmlC; dTDP-4-dehydrorhamnose 3,5-epimerase [EC:5.1.3.13]
K01588	DMN	8.45E-05	1.51E-05	4.09E-05	0.00784687	ko09100	ko09104	ko00230	purE; 5-(carboxyamino)imidazole ribonucleotide mutase [EC:5.4.99.18]
K01770	DMN	9.21E-05	2.46E-05	0.0017634	0.05422609	ko09100	ko09109	ko00900	ispF; 2-C-methyl-D-erythritol 2,4-cyclodiphosphate synthase [EC:4.6.1.12]
K01126	DMN	9.73E-05	2.40E-05	0.0008601	0.032443	ko09100	ko09103	ko00564	E3.1.4.46, glpQ, ugpQ; glycerophosphoryl diester phosphodiesterase [EC:3.1.4.46]
K01897	DMN	0.00010644	3.21E-05	0.0036905	0.09915716	ko09100	ko09103	ko00061	ACSL, fadD; long-chain acyl-CoA synthetase [EC:6.2.1.3]
K00330	DMN	0.000120397	3.70E-05	0.0038525	0.10201243	ko09100	ko09102	ko00190	nuoA; NADH-quinone oxidoreductase subunit A [EC:7.1.1.2]
K01785	DMN	0.000140475	1.92E-05	1.65E-06	0.00132775	ko09100	ko09101	ko00010	galM, GALM; aldose 1-epimerase [EC:5.1.3.3]
K00950	DMN	0.00014313	4.14E-05	0.0032492	0.08919197	ko09100	ko09108	ko00790	folK; 2-amino-4-hydroxy-6-hydroxymethyldihydropteridine diphosphokinase [EC:2.7.6.3]
K01187	DMN	0.000146358	4.26E-05	0.0032926	0.08967918	ko09100	ko09101	ko00052	malZ; alpha-glucosidase [EC:3.2.1.20]
K12373	DMN	0.000181586	5.90E-05	0.0064207	0.15879225	ko09100	ko09101	ko00520	HEXA_B; hexosaminidase [EC:3.2.1.52]
K01190	DMN	0.000252954	8.46E-05	0.0082592	0.19517752	ko09100	ko09101	ko00052	lacZ; beta-galactosidase [EC:3.2.1.23]

Supplementary table 2. List of differently expressed genes in DMN associated with carbohydrate, protein and lipid metabolisms.

Description	KEGG orthology	Coef.	SE	p-value	q-value
Carbohydrate metabolism (ko09101)					
galM, GALM; aldose 1-epimerase [EC:5.1.3.3]	K01785	0.00014	1.92E-05	1.65E-06	0.001328
UXS1, uxs; UDP-glucuronate decarboxylase [EC:4.1.1.35]	K08678	4.70E-05	1.01E-05	3.56E-06	0.002213
manB; phosphomannomutase [EC:5.4.2.8]	K01840	3.82E-05	8.00E-06	6.41E-06	0.003054
leuA, IMS; 2-isopropylmalate synthase [EC:2.3.3.13]	K01649	3.68E-05	8.87E-06	4.08E-05	0.007847
ldhA; D-lactate dehydrogenase [EC:1.1.1.28]	K03778	3.41E-05	8.31E-06	5.87E-05	0.009637
E3.2.1.1A; alpha-amylase [EC:3.2.1.1]	K07405	3.36E-05	8.43E-06	7.58E-05	0.010175
E2.2.1.2, talA, talB; transaldolase [EC:2.2.1.2]	K00616	4.31E-05	1.10E-05	9.45E-05	0.010259
yihX; glucose-1-phosphatase [EC:3.1.3.10]	K20866	4.45E-05	1.14E-05	9.66E-05	0.010259
galK; galactokinase [EC:2.7.1.6]	K00849	3.60E-05	9.52E-06	0.000157	0.013683
glyA, SHMT; glycine hydroxymethyltransferase [EC:2.1.2.1]	K00600	3.40E-05	9.01E-06	0.000171	0.013987
cimA; (R)-citramalate synthase [EC:2.3.1.182]	K09011	3.21E-05	8.42E-06	0.000174	0.014037
araA; L-arabinose isomerase [EC:5.3.1.4]	K01804	3.17E-05	8.32E-06	0.000186	0.014592
UGDH, ugd; UDPglucose 6-dehydrogenase [EC:1.1.1.22]	K00012	3.78E-05	1.03E-05	0.000238	0.016664
gpmI; 2,3-bisphosphoglycerate-independent phosphoglycerate mutase [EC:5.4.2.12]	K15633	3.57E-05	9.75E-06	0.000246	0.016792
GLDC, gcvP; glycine dehydrogenase [EC:1.4.4.2]	K00281	2.99E-05	7.44E-06	0.000268	0.017112
E4.1.1.15, gadB, gadA, GAD; glutamate decarboxylase [EC:4.1.1.15]	K01580	3.37E-05	9.23E-06	0.000267	0.017112
rpe, RPE; ribulose-phosphate 3-epimerase [EC:5.1.3.1]	K01783	4.75E-05	1.29E-05	0.000276	0.017112
ACSS1_2, acs; acetyl-CoA synthetase [EC:6.2.1.1]	K01895	2.98E-05	7.98E-06	0.00031	0.017769
murQ; N-acetylmuramic acid 6-phosphate etherase [EC:4.2.1.126]	K07106	3.43E-05	9.80E-06	0.000458	0.022231
leuB, IMDH; 3-isopropylmalate dehydrogenase [EC:1.1.1.85]	K00052	3.54E-05	1.02E-05	0.000541	0.024343
buk; butyrate kinase [EC:2.7.2.7]	K00929	3.39E-05	9.94E-06	0.000644	0.027487
PGK, pgk; phosphoglycerate kinase [EC:2.7.2.3]	K00927	3.21E-05	9.42E-06	0.000666	0.028151

korA, oorA, oforA; 2-oxoglutarate/2-oxoacid ferredoxin oxidoreductase subunit alpha [EC:1.2.7.3 1.2.7.11]	K00174	7.19E-05	1.75E-05	0.000777	0.030629
por, nifJ; pyruvate-ferredoxin/flavodoxin oxidoreductase [EC:1.2.7.1 1.2.7.-]	K03737	3.05E-05	9.08E-06	0.000811	0.031626
hprA; glycerate dehydrogenase [EC:1.1.1.29]	K00018	3.12E-05	9.31E-06	0.000821	0.031687
E1.1.1.40, maeB; malate dehydrogenase (oxaloacetate-decarboxylating)(NADP+) [EC:1.1.1.40]	K00029	2.90E-05	8.63E-06	0.00088	0.032491
murA; UDP-N-acetylglucosamine 1-carboxyvinyltransferase [EC:2.5.1.7]	K00790	3.45E-05	1.06E-05	0.0011	0.038593
gcvT, AMT; aminomethyltransferase [EC:2.1.2.10]	K00605	3.13E-05	9.64E-06	0.00119	0.040914
MUT; methylmalonyl-CoA mutase [EC:5.4.99.2]	K01847	6.56E-05	1.71E-05	0.001206	0.041289
GPI, pgi; glucose-6-phosphate isomerase [EC:5.3.1.9]	K01810	3.25E-05	1.02E-05	0.001454	0.046763
AMY, amyA, malS; alpha-amylase [EC:3.2.1.1]	K01176	2.55E-05	7.44E-06	0.001466	0.046997
murB; UDP-N-acetylmuramate dehydrogenase [EC:1.3.1.98]	K00075	3.30E-05	1.05E-05	0.001688	0.052732
abfA; alpha-L-arabinofuranosidase [EC:3.2.1.55]	K01209	4.23E-05	1.34E-05	0.001761	0.054226
E4.2.1.2A, fumA, fumB; fumarate hydratase, class I [EC:4.2.1.2]	K01676	3.12E-05	1.01E-05	0.001933	0.05863
mdh; malate dehydrogenase [EC:1.1.1.37]	K00024	5.97E-05	1.66E-05	0.002092	0.06256
PRPS, prsA; ribose-phosphate pyrophosphokinase [EC:2.7.6.1]	K00948	3.18E-05	1.04E-05	0.002151	0.062875
yqhD; NADP-dependent alcohol dehydrogenase [EC:1.1.-.-]	K08325	2.52E-05	8.13E-06	0.002207	0.063937
E3.2.1.22B, galA, rafA; alpha-galactosidase [EC:3.2.1.22]	K07407	6.54E-05	1.89E-05	0.002962	0.083039
malQ; 4-alpha-glucanotransferase [EC:2.4.1.25]	K00705	2.85E-05	9.63E-06	0.003102	0.085829
nagB, GNPDA; glucosamine-6-phosphate deaminase [EC:3.5.99.6]	K02564	7.43E-05	2.15E-05	0.00311	0.085829
malZ; alpha-glucosidase [EC:3.2.1.20]	K01187	0.000146	4.26E-05	0.003293	0.089679
deoC, DERA; deoxyribose-phosphate aldolase [EC:4.1.2.4]	K01619	3.85E-05	1.35E-05	0.004667	0.122139
ackA; acetate kinase [EC:2.7.2.1]	K00925	2.88E-05	1.04E-05	0.005597	0.142462
glgA; starch synthase [EC:2.4.1.21]	K00703	3.18E-05	1.14E-05	0.005662	0.143057
leuC, IPMI-L; 3-isopropylmalate/(R)-2-methylmalate dehydratase large subunit [EC:4.2.1.33 4.2.1.35]	K01703	2.97E-05	1.08E-05	0.005948	0.149216
HEXA_B; hexosaminidase [EC:3.2.1.52]	K12373	0.000182	5.90E-05	0.006421	0.158792
aldA; lactaldehyde dehydrogenase / glycolaldehyde dehydrogenase [EC:1.2.1.22 1.2.1.21]	K07248	2.13E-05	5.95E-06	0.007044	0.171765
katE, CAT, catB, srpA; catalase [EC:1.11.1.6]	K03781	2.46E-05	3.65E-06	0.00756	0.182667

pycB; pyruvate carboxylase subunit B [EC:6.4.1.1]	K01960	4.28E-05	1.43E-05	0.008012	0.190933
lacZ; beta-galactosidase [EC:3.2.1.23]	K01190	0.000253	8.46E-05	0.008259	0.195178
sdhA, frdA; succinate dehydrogenase / fumarate reductase, flavoprotein subunit [EC:1.3.5.1 1.3.5.4]	K00239	2.47E-05	8.74E-06	0.00862	0.201316
ENO, eno; enolase [EC:4.2.1.11]	K01689	3.15E-05	1.19E-05	0.008605	0.201316
TPI, tpiA; triosephosphate isomerase (TIM) [EC:5.3.1.1]	K01803	3.16E-05	1.21E-05	0.009248	0.212666
PK, pyk; pyruvate kinase [EC:2.7.1.40]	K00873	3.06E-05	1.20E-05	0.011369	0.249931
Protein metabolism (ko09105)					
argD; acetylornithine/N-succinyldiaminopimelate aminotransferase [EC:2.6.1.11 2.6.1.17]	K00821	5.38E-05	9.90E-06	5.46E-08	0.000143
kbl, GCAT; glycine C-acetyltransferase [EC:2.3.1.29]	K00639	4.82E-05	9.59E-06	5.15E-07	0.00054
argB; acetylglutamate kinase [EC:2.7.2.8]	K00930	4.31E-05	1.03E-05	2.79E-05	0.0075
hutH, HAL; histidine ammonia-lyase [EC:4.3.1.3]	K01745	3.89E-05	9.27E-06	2.74E-05	0.0075
hisD; histidinol dehydrogenase [EC:1.1.1.23]	K00013	3.74E-05	8.88E-06	3.02E-05	0.007669
ald; alanine dehydrogenase [EC:1.4.1.1]	K00259	2.83E-05	7.88E-06	0.000469	0.022649
alr-murF; Alr-MurF fusion protein [EC:5.1.1.1 6.3.2.10]	K01798	3.40E-05	8.25E-06	5.53E-05	0.009501
hisA; phosphoribosylformimino-5-aminoimidazole carboxamide ribotide isomerase [EC:5.3.1.16]	K01814	4.12E-05	1.04E-05	7.21E-05	0.010083
murF; UDP-N-acetylmuramoyl-tripeptide--D-alanyl-D-alanine ligase [EC:6.3.2.10]	K01929	3.67E-05	9.36E-06	9.11E-05	0.010175
hisG; ATP phosphoribosyltransferase [EC:2.4.2.17]	K00765	3.47E-05	9.12E-06	0.00015	0.013683
aroC; chorismate synthase [EC:4.2.3.5]	K01736	3.55E-05	9.38E-06	0.000155	0.013683
nadB; L-aspartate oxidase [EC:1.4.3.16]	K00278	3.29E-05	9.18E-06	0.000343	0.018871
hisB; imidazoleglycerol-phosphate dehydratase / histidinol-phosphatase [EC:4.2.1.19 3.1.3.15]	K01089	3.11E-05	8.56E-06	0.000319	0.018149
metH, MTR; 5-methyltetrahydrofolate--homocysteine methyltransferase [EC:2.1.1.13]	K00548	3.04E-05	8.14E-06	0.000261	0.017112
metK; S-adenosylmethionine synthetase [EC:2.5.1.6]	K00789	3.73E-05	1.14E-05	0.001149	0.039905
aspB; aspartate aminotransferase [EC:2.6.1.1]	K00812	5.09E-05	1.80E-05	0.011282	0.249157
argE; acetylornithine deacetylase [EC:3.5.1.16]	K01438	3.34E-05	9.50E-06	0.000437	0.021598
E2.6.1.42, ilvE; branched-chain amino acid aminotransferase [EC:2.6.1.42]	K00826	2.99E-05	1.00E-05	0.002878	0.080897
pheA2; prephenate dehydratase [EC:4.2.1.51]	K04518	3.56E-05	1.02E-05	0.000489	0.023003

hisF; imidazole glycerol-phosphate synthase subunit HisF [EC:4.3.2.10]	K02500	3.38E-05	9.75E-06	0.000532	0.024343
kamA; lysine 2,3-aminomutase [EC:5.4.3.2]	K01843	3.03E-05	8.72E-06	0.000543	0.024345
hisC; histidinol-phosphate aminotransferase [EC:2.6.1.9]	K00817	4.52E-05	1.30E-05	0.000559	0.024846
E3.5.1.1, ansA, ansB; L-asparaginase [EC:3.5.1.1]	K01424	5.61E-05	1.95E-05	0.010962	0.244573
murE; UDP-N-acetylmuramoyl-L-alanyl-D-glutamate--2,6-diaminopimelate ligase [EC:6.3.2.13]	K01928	3.34E-05	9.72E-06	0.000582	0.025567
speA; arginine decarboxylase [EC:4.1.1.19]	K01585	3.49E-05	1.02E-05	0.000642	0.027487
hutI, AMDHD1; imidazolonepropionase [EC:3.5.2.7]	K01468	2.88E-05	8.33E-06	0.00071	0.029207
dapF; diaminopimelate epimerase [EC:5.1.1.7]	K01778	3.51E-05	1.05E-05	0.000841	0.03205
proA; glutamate-5-semialdehyde dehydrogenase [EC:1.2.1.41]	K00147	3.39E-05	1.02E-05	0.000876	0.032468
dapB; 4-hydroxy-tetrahydrodipicolinate reductase [EC:1.17.1.8]	K00215	3.75E-05	1.13E-05	0.000917	0.033751
speG, SAT; diamine N-acetyltransferase [EC:2.3.1.57]	K00657	4.32E-05	1.30E-05	0.000976	0.035453
aroB; 3-dehydroquinate synthase [EC:4.2.3.4]	K01735	3.15E-05	9.59E-06	0.001042	0.037177
glsA, GLS; glutaminase [EC:3.5.1.2]	K01425	4.65E-05	1.28E-05	0.000346	0.018902
trpB; tryptophan synthase beta chain [EC:4.2.1.20]	K06001	3.09E-05	9.52E-06	0.001157	0.039912
asd; aspartate-semialdehyde dehydrogenase [EC:1.2.1.11]	K00133	3.26E-05	1.06E-05	0.002021	0.061087
proB; glutamate 5-kinase [EC:2.7.2.11]	K00931	3.33E-05	1.08E-05	0.00214	0.062875
hisH; imidazole glycerol-phosphate synthase subunit HisH [EC:4.3.2.10]	K02501	3.52E-05	1.17E-05	0.002686	0.076535
ilvD; dihydroxy-acid dehydratase [EC:4.2.1.9]	K01687	3.55E-05	1.00E-05	0.000407	0.021104
argC; N-acetyl-gamma-glutamyl-phosphate reductase [EC:1.2.1.38]	K00145	3.06E-05	1.06E-05	0.004064	0.107346
hutU, UROC1; urocanate hydratase [EC:4.2.1.49]	K01712	2.45E-05	8.94E-06	0.006234	0.155638
lysC; aspartate kinase [EC:2.7.2.4]	K00928	5.08E-05	1.66E-05	0.006696	0.164043
argH, ASL; argininosuccinate lyase [EC:4.3.2.1]	K01755	3.45E-05	1.00E-05	0.000576	0.025474
asnB, ASNS; asparagine synthase (glutamine-hydrolysing) [EC:6.3.5.4]	K01953	2.64E-05	1.01E-05	0.008911	0.205825
NIT2, yafV; omega-amidase [EC:3.5.1.3]	K13566	3.50E-05	7.91E-06	3.99E-05	0.007847
Lipid metabolism (ko09103)					
MCH; medium-chain acyl-[acyl-carrier-protein] hydrolase [EC:3.1.2.21]	K01071	3.76E-05	8.42E-06	9.84E-06	0.003968

psd, PISD; phosphatidylserine decarboxylase [EC:4.1.1.65]	K01613	4.19E-05	9.87E-06	2.18E-05	0.007141
fabI; enoyl-[acyl-carrier protein] reductase I [EC:1.3.1.9 1.3.1.10]	K00208	3.33E-05	8.64E-06	0.000137	0.012914
gpsA; glycerol-3-phosphate dehydrogenase (NAD(P)+) [EC:1.1.1.94]	K00057	3.38E-05	9.42E-06	0.000338	0.018778
lpxC-fabZ; UDP-3-O-[3-hydroxymyristoyl] N-acetylglucosamine deacetylase / 3-hydroxyacyl-[acyl-carrier-protein] dehydratase [EC:3.5.1.108 4.2.1.59]	K16363	3.10E-05	8.78E-06	0.000432	0.021598
fabH; 3-oxoacyl-[acyl-carrier-protein] synthase III [EC:2.3.1.180]	K00648	4.57E-05	1.33E-05	0.000672	0.028317
fabD, MCAT, MCT1; [acyl-carrier-protein] S-malonyltransferase [EC:2.3.1.39]	K00645	3.35E-05	9.90E-06	0.000705	0.029102
SRD5A1; 3-oxo-5-alpha-steroid 4-dehydrogenase 1 [EC:1.3.1.22]	K12343	3.13E-05	9.26E-06	0.000749	0.030005
E3.1.4.46, glpQ, ugpQ; glycerophosphoryl diester phosphodiesterase [EC:3.1.4.46]	K01126	9.73E-05	2.40E-05	0.00086	0.032443
ACSL, fadD; long-chain acyl-CoA synthetase [EC:6.2.1.3]	K01897	0.000106	3.21E-05	0.00369	0.099157
fabF, OXSM, CEM1; 3-oxoacyl-[acyl-carrier-protein] synthase II [EC:2.3.1.179]	K09458	2.97E-05	1.07E-05	0.005654	0.143057
CHO1, pssA; CDP-diacylglycerol---serine O-phosphatidyltransferase [EC:2.7.8.8]	K17103	2.69E-05	1.01E-05	0.007668	0.183985
clsA_B; cardiolipin synthase A/B [EC:2.7.8.-]	K06131	5.24E-05	1.80E-05	0.008199	0.194502

Supplementary Figure 1. Rarefaction curves for species richness in each sample. Blue- and yellow-colored lines represent samples in DMN and control groups, respectively.

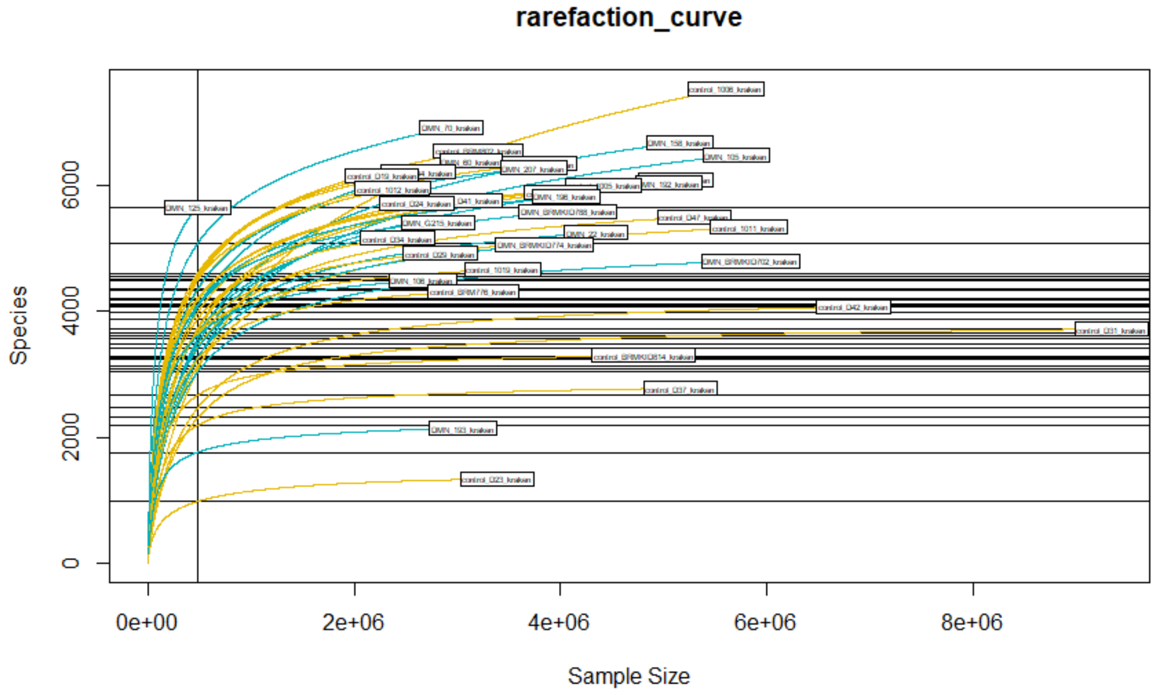

Supplement: Supplemental file 1 — Tables S1 and S2 and Fig. S1. Download spectrum.02344-22-s0001.pdf, PDF file, 0.9 MB [file spectrum.02344-22-s0001.pdf]
